# m^6^A-dependent glycolysis enhances colorectal cancer progression

**DOI:** 10.1186/s12943-020-01190-w

**Published:** 2020-04-03

**Authors:** Chaoqin Shen, Baoqin Xuan, Tingting Yan, Yanru Ma, Pingping Xu, Xianglong Tian, Xinyu Zhang, Yingying Cao, Dan Ma, Xiaoqiang Zhu, Youwei Zhang, Jing-Yuan Fang, Haoyan Chen, Jie Hong

**Affiliations:** 1 State Key Laboratory for Oncogenes and Related Genes; Key Laboratory of Gastroenterology & Hepatology, Ministry of Health; Division of Gastroenterology and Hepatology; Shanghai Cancer Institute; Shanghai Institute of Digestive Disease; Renji Hospital, Shanghai Jiao Tong University School of Medicine, 145 Middle Shandong Road, Shanghai, 200001 China; 2 Department of Medical Oncology, Xuzhou Central Hospital, Xuzhou Medical University, Xuzhou, 221009 China

**Keywords:** m^6^A modification, METTL3, Glycolysis, Colorectal cancer, HK2, GLUT1

## Abstract

**Background:**

Epigenetic alterations are involved in various aspects of colorectal carcinogenesis. *N*^6^-methyladenosine (m^6^A) modifications of RNAs are emerging as a new layer of epigenetic regulation. As the most abundant chemical modification of eukaryotic mRNA, m^6^A is essential for the regulation of mRNA stability, splicing, and translation. Alterations of m^6^A regulatory genes play important roles in the pathogenesis of a variety of human diseases. However, whether this mRNA modification participates in the glucose metabolism of colorectal cancer (CRC) remains uncharacterized.

**Methods:**

Transcriptome-sequencing and liquid chromatography-tandem mass spectrometry (LC-MS) were performed to evaluate the correlation between m^6^A modifications and glucose metabolism in CRC. Mass spectrometric metabolomics analysis, in vitro and in vivo experiments were conducted to investigate the effects of METTL3 on CRC glycolysis and tumorigenesis. RNA MeRIP-sequencing, immunoprecipitation and RNA stability assay were used to explore the molecular mechanism of METTL3 in CRC.

**Results:**

A strong correlation between METTL3 and ^18^F-FDG uptake was observed in CRC patients from Xuzhou Central Hospital. METTL3 induced-CRC tumorigenesis depends on cell glycolysis in multiple CRC models. Mechanistically, METTL3 directly interacted with the 5′/3’UTR regions of *HK2*, and the 3’UTR region of *SLC2A1* (GLUT1), then further stabilized these two genes and activated the glycolysis pathway. M^6^A-mediated *HK2* and *SLC2A1* (GLUT1) stabilization relied on the m^6^A reader IGF2BP2 or IGF2BP2/3, respectively.

**Conclusions:**

METTL3 is a functional and clinical oncogene in CRC. METTL3 stabilizes *HK2* and *SLC2A1* (GLUT1) expression in CRC through an m^6^A-IGF2BP2/3- dependent mechanism. Targeting METTL3 and its pathway offer alternative rational therapeutic targets in CRC patients with high glucose metabolism.

## Background

As the fourth most common malignancies and the third leading cause of cancer deaths worldwide [1], colorectal cancer (CRC) is a major cause of morbidity and mortality in the world. Despite that the substantial diagnostic and therapeutic strategies have been improved, the survival time of CRC patients have been increased in recent years, the mortality rate of colorectal cancer remains high [2]. The lack of effective interventions and precise biomarkers of the cancer urgently need a better understanding of the molecular mechanism of CRC initiation and progression.

An emerging hallmark of invasive cancer cells is energy metabolism, which includes elevated glycolysis activation and higher lactate fermentation known as the Warburg effect [3–5]. Targeting lactate production, which is the final product of glycolysis, is regarded to be a promising therapeutic approach in cancer [6]. Recent studies found that inhibition of EGFR signaling resulted in dramatically lung cancer reduction by reversing of Warburg effect and reactivation of oxidative phosphorylation [7], which highlights the perspective role for the therapeutic target of glycolysis. However, the molecular basis for glycolysis and its role in cancer growth remain unclear.

*N*^6^-methyladenosine (m^6^A) modification is the most abundant posttranscriptional internal mRNA modification, which mediates various biological processes in eukaryotes [8, 9]. m^6^A RNA modification is usually enriched near stop codons and terminal exons in over 25% of human transcripts [10, 11]. In mammals, m^6^A, which is dynamic and reversible RNA modification in mammalian cells, is post-transcriptionally installed by a m^6^A methyltransferase complex that contains an enzymatic subunit METTL3, and its assistant co-factors METTL14 and WTAP within the consensus motif of G(m^6^A) C (70%) or A(m^6^A) C (30%) [12–14]. And this mRNA modification can be removed by demethylases FTO and ALKBH5 [15, 16]. Recent studies have shown that m^6^A modification in mRNAs participates in RNA metabolisms to regulate mRNA stability, splicing, transport, localization, as well as in stem cell self-renewal and differentiation, tissue development in normal cellular physiological and disease status [10, 17–19]. Recent studies have reported that the writers, readers, and erasers of m^6^A may play important roles in various cancer initiation, progression and response to immunotherapy via different regulation patterns [18, 20–24]. Despite these recent discoveries between m^6^A modification with malignant cancer development and treatment, the status of m^6^A modification and the underlying regulatory mechanism in CRC, especially in the glycolytic metabolism of CRC remains little known. In the current study, we investigated the role of m^6^A modification in CRC and identified the oncogenic role of METTL3, with a biological, mechanistic, and clinical impact on human CRC and glucose metabolism. Our findings on the implication of METTL3-mediated m^6^A modification in epigenetic stabilizing of *HK2* and *SLC2A1* (GLUT1) illustrated the critical role of m^6^A epitranscriptomic change in human colorectal carcinogenesis and glycolysis pathways.

## Methods

### Patient specimens

We used three Cohorts of patients with colorectal cancer who underwent surgery between 2012 and 2019. Cohort 1 (fresh tissues and paraffin-embedded tissues) were from Xuzhou Central Hospital, Xuzhou Medical University; Cohort 2 (paraffin-embedded tissues) were from the Eastern Campus of Renji Hospital, Shanghai Jiao Tong University School of Medicine; Cohort 3 (fresh tissues) were from the Western Campus of Renji Hospital, Shanghai Jiao Tong University School of Medicine. The study protocol was approved by the ethics committee of Shanghai Jiao Tong University School of Medicine. Written informed consent was obtained from all participants in this study. All the research was carried out in accordance with the provisions of the Declaration of Helsinki of 1975. None of these patients had received radiotherapy or chemotherapy prior to surgery.

### M^6^A dot blot and m^6^A quantification

Polyadenylated mRNA was purified by GenElute™ mRNA Miniprep Kit (Sigma, St. Louis, MO) from previously isolated total RNA. The m^6^A dot blot assay was performed as previously described [25]. The global m^6^A levels in mRNA were measured with EpiQuik m^6^A RNA Methylation Quantification Kit (Colorimetric) (Epigentek, Farmingdale, NY) following the manufacturer’s protocol. The detailed m^6^A dot blot and m^6^A quantification protocols were described in the Supplementary Materials and Methods.

### MeRIP and MeRIP-qPCR

The m^6^A-immunoprecipitation and library preparation was performed according to a published protocol [10]. Real-time PCR was carried out following m^6^A-IP to quantify the changes to m^6^A methylation of a certain target gene. The detailed MeRIP and MeRIP-qPCR protocols were described in the Supplementary Materials and Methods.

### Glucose uptake, lactate production, hexokinase activity assay, seahorse metabolic analysis

Glucose Uptake, L-Lactate Colorimetric assay, Hexokinase Colorimetric assay, Seahorse XF Glycolysis Stress Test and Seahorse XF Cell Mito Stress Test were detailed described in the Supplementary Materials and Methods.

### RNA Immunoprecipitation

RNA Immunoprecipitation (RIP) assays were conducted using the Magna RIP Kit (Millipore, New Bedford, MA) and the detailed protocol was described in the Supplementary Materials and Methods.

### Statistical analysis

Statistical analyses were carried out using the program R (www.r-project.org). Recurrence-free survival was evaluated by Kaplan-Meier survival curve and Log-rank tests. Statistical significance was assessed by unpaired two-tailed Student’s-tests. Single-sample gene set enrichment analysis (ssGSEA) was used to assess gene set activation scores in gene expression profiling data. ssGSEA calculates a sample level gene set score by comparing the distribution of gene expression ranks inside and outside the gene set. The ssGSEA score was calculated by Gene Set Variation Analysis (GSVA) R package. Data were examined to determine whether they were normally distributed with the One-Sample Kolmogorov-Smirnov test. If the data were normally distributed, comparisons of measurement data between two groups were performed using independent sample t test and the comparisons among three or more groups were first performed by one-way ANOVA test. If the results showed significant difference, when the data were skewed distribution, comparisons were performed by nonparametric test. Measurement data between two groups were performed using nonparametric Mann-Whitney test.

### Data availability

The raw sequencing data have been deposited in the Gene Expression Omnibus database under the accession number GSE130012. All the other data generated in this study are included in the article and the additional files.

More detailed materials and methods are in the Supplementary Methods.

## Results

### METTL3 is closely correlated with glycolysis in colorectal cancer

**Fig. 1:**
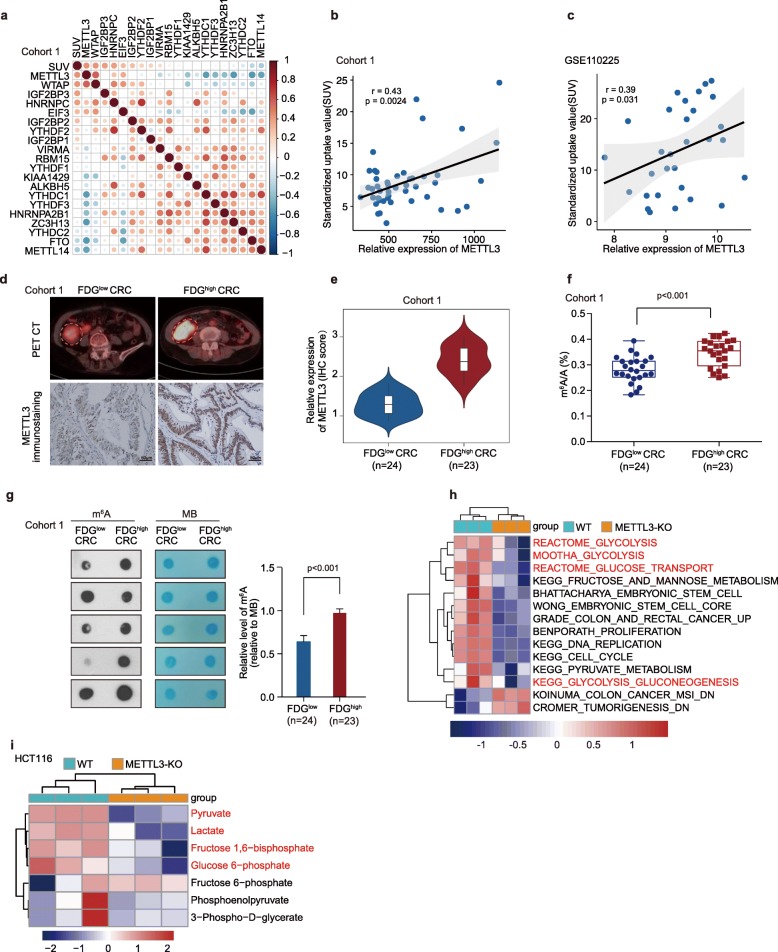
METTL3 is closely correlated with glycolytic metabolism in CRC. (**a**) The correlation matrix showed the relationship between the expression of m^6^A genes and SUV_max_ (FDG maximal standardized uptake value) in 47 CRC patients of Cohort 1. (**b**) Correlation between FDG uptake (SUV_max_) and METTL3 expression in 47 CRC patients of Cohort 1. (**c**) Correlation between FDG uptake (SUV_max_) and METTL3 expression in 30 CRC patients of another independent cohort (GSE110225). (**d**) Representative PET/CT images and METTL3 immunohistochemical images of CRC patients with FDG high uptake and FDG low uptake in Cohort 1. (**e**) Statistical analysis of METTL3 expression in CRC patients with FDG high uptake and patients with FDG low uptake, nonparametric Mann-Whitney test. (**f**) LC-MS/MS quantification of the m^6^A/A ratio in poly(A) RNA isolated from CRC patients with FDG high uptake or FDG low uptake in Cohort 1, nonparametric Mann-Whitney test. (**g**) Representative images and relative quantitative information of m^6^A dot blot assay showed global m^6^A abundance in CRC patients with FDG high uptake or FDG low uptake in Cohort 1. MB, methylene blue staining (as a loading control). (**h**) ssGSEA analysis was performed to show the pathways closely correlated with METTL3 expression levels in CRC cells; *n* = 3. (**i**) LC-MS/MS-based analysis showed the major metabolites altered in the glycolytic pathway in HCT116 METTL3-KO cells relative to WT cells. The differentially expressed metabolites (*p* < 0.05) were highlighted in red; *n* = 3. (WT, wild type; METTL3-KO, METTL3-knockout)

To explore the correlation between m^6^A modifications with glycolysis metabolism in colorectal cancer (CRC), real-time PCR analysis was performed to compare the regulated-m^6^A gene expression profiles in 47 CRC patients who had been imaged for ^18^F-FDG PET (Cohort 1). We observed that there was a most significant correlation between FDG uptake and METTL3 expression in CRC patients (Fig. 1a-b), as measured by FDG maximal standardized uptake value (SUV_max_). In addition, we observed that there was a significant correlation between FDG uptake and METTL3 expression in an independent CRC patients’ dataset (GSE110225), as measured by SUV_max_ (Fig. 1c). Further analysis revealed that a significant correlation between FDG uptake and METTL3 immunohistochemical staining existed in CRC patients of Cohort 1 (Fig. 1d-e). In line with METTL3 higher expression in CRC patients with vigorous glucose metabolism, LC-MS/MS (liquid chromatography-tandem mass spectrometry) (Fig. 1f) and m^6^A dot blot (Fig. 1g) experiments revealed that the m^6^A modification level was significantly increased in these CRC patients with higher FDG uptake as well. To elucidate whether METTL3 plays a role in CRC glycolysis and tumorigenesis, we then performed RNA-seq analysis to compare the gene expression profiles of METTL3-knockout and wild type (WT) HCT116 CRC cells, which exhibit higher METTL3 expression (Figure S1a). METTL3 knockout of CRISPR-cas9 generated cell was confirmed by sequencing and Western blot analysis (Figure S1b-c). A total of 2848 downregulated genes and 3046 upregulated genes (*p* < 0.05) were detected (raw data accessible via GEO number GSE130012) after knockout of METTL3 in HCT116 cells (Table S1). Gene set enrichment analysis (GSEA) of RNA-seq data showed that the gene signatures of “Glucose uptake” were enriched in HCT116 WT cells (Figure S1d). Single-sample GSEA (ssGSEA) [26] revealed that the gene sets related to Reactome_Glycolysis, Mootha_Glycolysis, Reactome_Glucose_Transport and KEGG_Glycolysis_Gluconeogenesis (glycolysis pathway) and Grade_Colon_And_Rectal_Cancer_Up (colorectal-carcinogenesis signature) were negatively correlated with METTL3-knockout CRC cells (Fig. 1h, Table S2). Besides, further mass spectrometric metabolomics analysis [27] was performed in HCT116 WT and METTL3-knockout cells. The high through put metabolic analysis showed that knockout of METTL3 significantly reduced the levels of key components of glycolysis pathway, such as glucose-6-phosphate, fructose 1,6-bisphosphate, pyruvate and lactate in HCT116 cells (Fig. 1i and Table S3). These data indicate that METTL3 may mediate glycolytic metabolism and carcinogenesis in CRC patients.

### METTL3 drives glycolytic metabolism in colorectal cancer

**Fig. 2:**
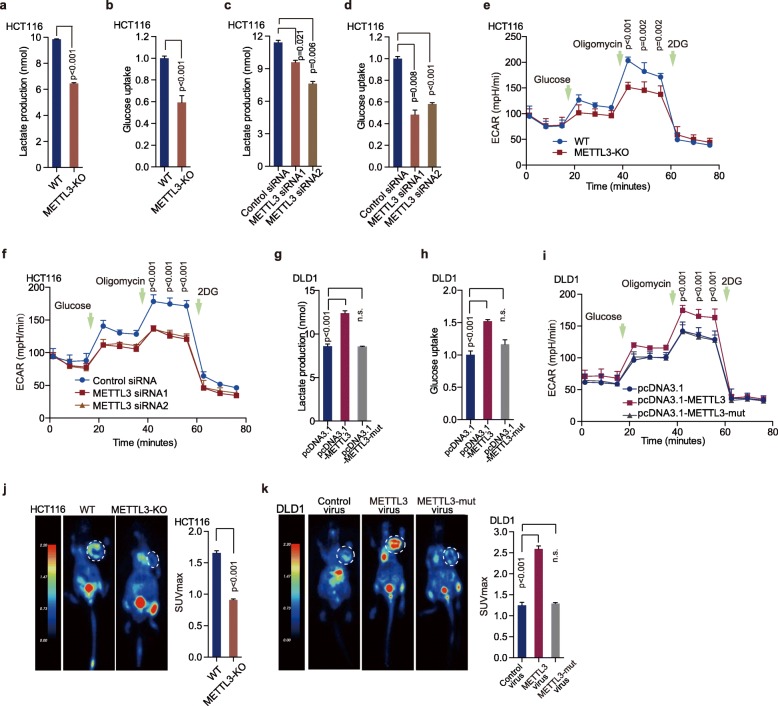
METTL3 drives glycolytic metabolism in CRC. (**a-b**) Lactate production (**a**), glucose uptake (**b**) were measured in HCT116 WT and METTL3-KO cells by colorimetric analysis, *n* = 3, nonparametric Mann–Whitney test. (**c-d**) Lactate production (**c**) and glucose uptake (**d**) were measured in HCT116 CRC cells transfected with control siRNA and METTL3 siRNA1/2 by colorimetric analysis; *n* = 3, nonparametric Mann–Whitney test. (**e-f**) ECAR was detected in HCT116 WT, METTL3- knockout cells (**e**) and HCT116 CRC cells transfected with control siRNA or METTL3 siRNA1/2 (**f**), *n* = 4, nonparametric Mann–Whitney test. (**g-i**) Lactate production (**g**), glucose uptake (**h**) and ECAR (**i**) were assessed in DLD1 cells transfected with pcDNA3.1, pcDNA3.1-METTL3 and pcDNA3.1-METTL3-mut, *n* = 3, nonparametric Mann–Whitney test. (**j**) Representative images of ^18^F-FDG uptake by micro-PET imaging in WT and METTL3-KO xenograft mouse models. White circles indicated tumor glucose uptake. Maximum uptake values (SUV_max_) for xenografts measured by FDG-PET were presented, nonparametric Mann–Whitney test. (**k**) Representative images of ^18^F-FDG uptake by micro-PET imaging in control virus, overexpression of METTL3 or METTL3-mut virus xenograft mouse models. White circles indicated tumor glucose uptake. Maximum uptake values (SUV_max_) for xenografts measured by FDG-PET were presented, nonparametric Mann–Whitney test. Fig. 2k and Fig. 6k shared experimental controls and METTL3-overexpression group. (WT, wild type; METTL3-KO, METTL3-knockout; *n* = 3)

Further functional colorimetric validation showed that lactic acid production (a key metabolite of glycolysis) and glucose uptake were both significantly decreased after METTL3 knockout in HCT116 cells (Fig. 2a-b). Knockdown of METTL3 significantly reduced lactic acid production as well as glucose uptake in HCT116 cells (Fig. 2c-d, Figure S2a) and SW480 cells (Figure S2b-d). To figure out whether alteration of METTL3 directly influence glycolytic metabolism, we measured extracellular acidification rate (ECAR) and oxygen consumption rate (OCR) in CRC cells after manipulating METTL3. Knockout or knockdown of METTL3 significantly reduced ECAR levels in HCT116 (Fig. 2e-f) and SW480 cells (Figure S2e), compared with control cells. However, the deletion of METTL3 had no significant effect on OCR levels in HCT116 cells and SW480 cells (Figure S2f).

To elucidate whether METTL3-induced CRC glycolysis depends on its methyltransferase function, we constructed METTL3 wild-type (pcDNA3.1-METTL3) and mutant (pcDNA3.1-METTL3-mut, with MTase domain deletion) recombination plasmids (Figure S2g) [28]. Both of pcDNA3.1-METTL3 and pcDNA3.1-METTL3-mut were successfully overexpressed in DLD1 cells (Figure S2h-i). In gain of function assays, overexpression of METTL3 dramatically increased lactic acid production (Fig. 2g), glucose absorption (Fig. 2h) and ECAR levels (Fig. 2i) in DLD1 cells. Deletion of the MTase domain of METTL3 blocked METTL3-induced glycolysis in CRC cells (Fig. 2g-i). To further examine the effects of METTL3 on glycolysis, we used ^18^F-FDG PET ([^18^F]-fluoro-2-deoxyglucose positron emission tomography) to measure glucose uptake in vivo*.* Mouse PET-CT data showed that knockout of METTL3 significantly reduced glucose uptake in xenograft mouse tumor model (Fig. 2j). Overexpression of WT METTL3, but not METTL3-mut, dramatically increased glucose uptake in vivo (Fig. 2k). These data indicate that METTL3 regulates glycolytic metabolism in colorectal cancer via its methyltransferase domain.

### METTL3-induced proliferation depends on the activation of glycolysis in colorectal cancer

**Fig. 3:**
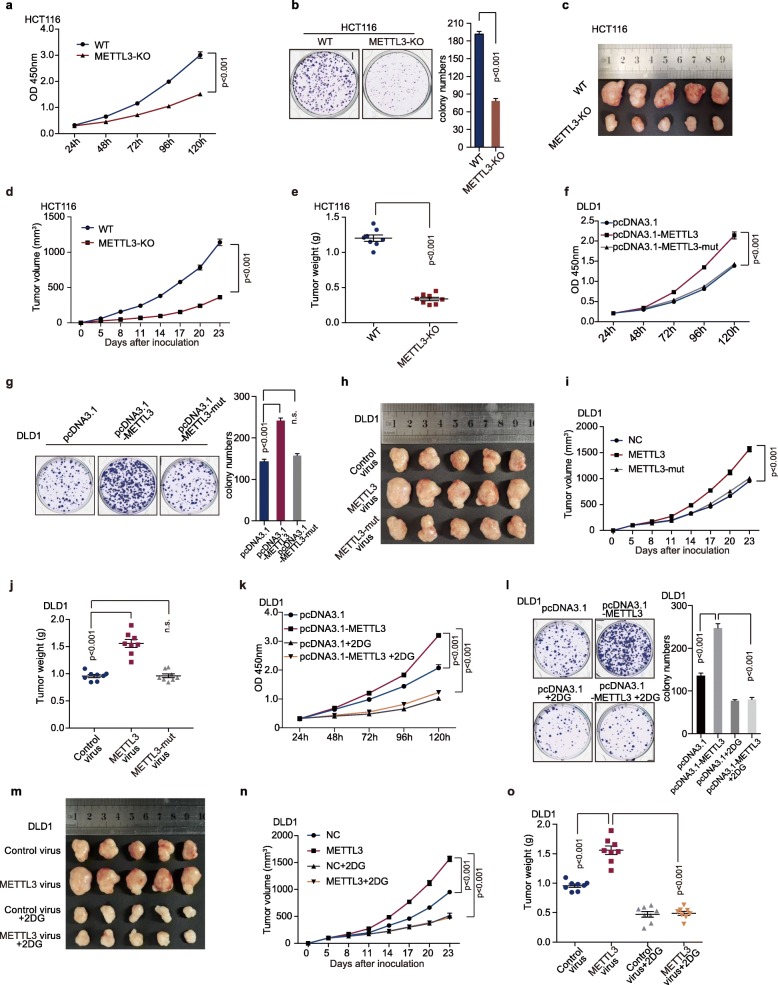
METTL3-induced cell survival depends on glycolytic metabolism. (**a**) Cell proliferation assay was performed by CCK8 assay in HCT116 WT or METTL3-KO cells, *n* = 6, nonparametric Mann–Whitney test. (**b**) Colony formation assay was performed in HCT116 WT or METTL3-KO cells, *n* = 3, nonparametric Mann–Whitney test. (**c**) Representative images of tumors in nude mice bearing HCT116 WT or METTL3-KO cells, *n* = 8. (**d-e**) Tumor volumes(**d**) and tumor weights (**e**) were measured in mice bearing HCT116 WT or METTL3-KO cells, *n* = 8, nonparametric Mann–Whitney test. (**f**) Cell proliferation of DLD-1 cells was measured by CCK8 assay after transfection of pcDNA3.1, pcDNA3.1-METTL3 and pcDNA3.1-METTL3-mut (METTL3 with MTase domain deletion), *n* = 6, nonparametric Mann–Whitney test. (**g**) Colony formation assay was performed in DLD1 cells after transfection with pcDNA3.1, pcDNA3.1-METTL3 and pcDNA3.1-METTL3-mut; *n* = 3, nonparametric Mann–Whitney test. (**h-j**) Representative images of tumors (**h**), statistical analysis of tumor volumes (**i**) and tumor weights (**j**) in nude mice bearing DLD1 cells in different groups (*n* = 8, nonparametric Mann–Whitney test). Fig 3h, Fig. 3m and Figure S6j shared experimental controls and METLL3-overexpression group. (**k-l**) Cell proliferation (**k**) and colony formation assay (**l**) were performed in DLD1 cells after transfection with control plasmids, METTL3 overexpression plasmids, and subsequently with 5 mM 2-DG (2-Deoxyglucose) treatment; nonparametric Mann–Whitney test. (**m-o**) Representative images of tumors (**m**), statistical analysis of tumor volumes (**n**) and tumor weights (**o**) in nude mice bearing DLD1 cells in different groups (*n* = 8, nonparametric Mann–Whitney test). Fig. 3h, Fig. 3m and Figure S6j shared experimental controls and METTL3-overexpression group. (WT, wild type; METTL3-KO, METTL3-knockout)

METTL3 has been reported as a pivotal modulator in tumorigenesis [29]; however, the role of METTL3 in CRC is paradoxical [30, 31]. GSEA of RNA-seq data showed that the gene signatures of CRC and DNA replication were enriched in HCT116 WT cells, compared with METTL3-knockout cells (Figure S3a-b). The top-scoring genes in the gene sets included key carcinogenesis genes, CDK1, PCNA, and CDCA7. Real-time PCR confirmed that knockout or knockdown of METTL3 expression dramatically reduced the expression of the key genes of tumorigenesis (Figure S3c-e). Our functional validation data showed that knockout or knockdown of METTL3 abolished cell proliferation (Fig. 3a, Figure S3f) and colony formation (Fig. 3b, Figure S3g) in HCT116 cells and SW480 cells (Figure S3h-i). Knockout of METTL3 dramatically reduced HCT116 tumor growth (Fig. 3c-d) and tumor weight (Fig. 3e) in xenograft mouse models.

In gain of function assays, overexpression of METTL3 increased cell proliferation (Fig. 3f) and colony formation (Fig. 3g) of DLD1 cells in vitro. Furthermore, overexpression of METTL3 dramatically increased DLD1 tumor growth (Fig. 3h-i) and tumor weight (Fig. 3j) in xenograft mouse models. However, the same phenomenon was not observed both in vitro (Fig. 3f-g) and in vivo (Fig. 3h-j) after overexpression of METTL3-mut. In addition, 2-DG (an inhibitor of glycolysis pathway) treatment significantly blocked METTL3-induced cell proliferation (Fig. 3k), and colony formation (Fig. 3l) in vitro and in vivo (Fig. 3m-o). There was no difference in tumor growth (Figure S3j-k) and tumor weight (Figure S3l) among different control groups. The data strongly suggests that METTL3 may be a tumor-driver gene and promote CRC progression by regulating colorectal cancer glucose metabolism.

### Transcriptome-wide m^6^A-seq and RNA-seq assays identified potential targets of METTL3 in colorectal cancer

**Fig. 4:**
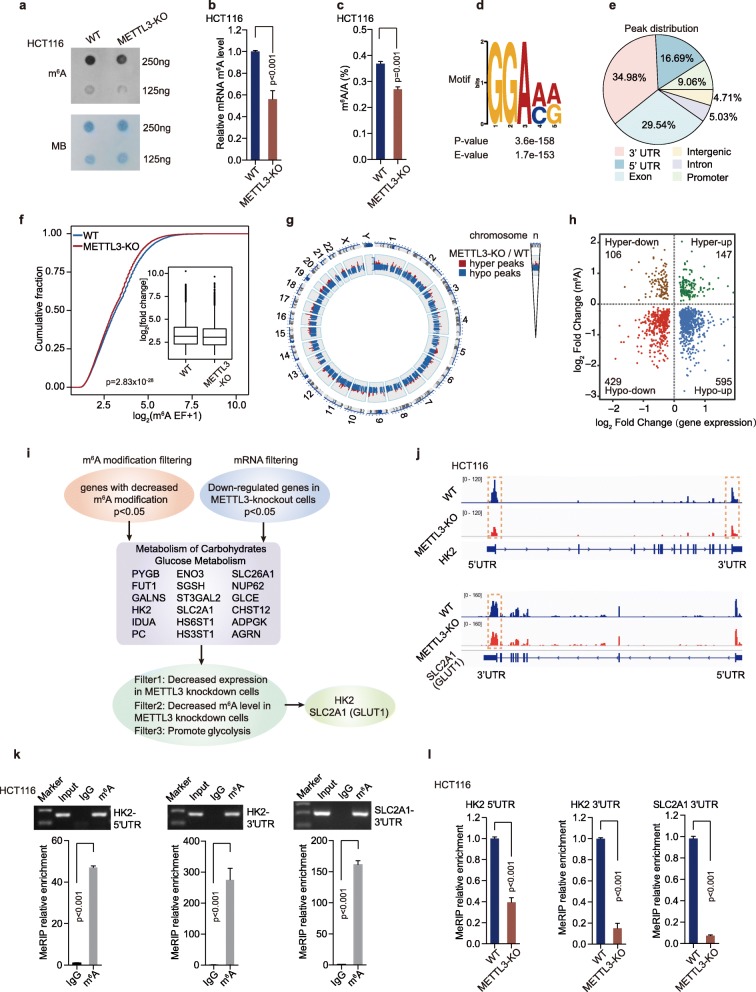
Transcriptome-wide m^6^A-seq and RNA-seq assays identified potential targets of METTL3 in colorectal cancer. (**a**) The m^6^A dot blot assay of global m^6^A abundance in mRNA of HCT116 WT and METTL3-knockout cells. MB, methylene blue staining (as a loading control). (**b**) The global m^6^A levels in mRNA of HCT116 WT and METTL3- knockout cells were measured by the EpiQuik™ m^6^A RNA Methylation Quantification Kit, *n* = 3, nonparametric Mann–Whitney test. (**c**) LC-MS/MS quantification of the m^6^A/A ratio in poly(A) RNA in HCT116 WT and METTL3-KO cells. (**d**) HOMER motif analysis revealed the top consensus m^6^A motif in HCT116 cells. (**e**) Graphs of m^6^A peak distribution illustrating the proportion of common m^6^A peaks in the indicated regions in HCT116 cells. (**f**) Cumulative curves of m^6^A abundance (log_2_(m^6^A EF + 1)) in HCT116 WT and METTL3-KO cells. The abundance of m^6^A immunoprecipitation was normalized to input when calculating the enrichment fold. *P* value was calculated using two-sided Wilcoxon and Mann–Whitney test. EF, enrichment folds. (**g**) Circos plot showing the distribution of hypermethylated (hyper) and hypomethylated (hypo) m^6^A peaks in the human transcriptome of HCT116 METTL3-KO cells compared with WT cells. (**h**) Distribution of genes with a significant change in both m^6^A level and gene expression level in HCT116 METTL3-KO cells compared with WT cells. (**i**) The flow chart for selected candidate target genes of METTL3 in HCT116 METTL3-KO cells is shown. (**j**) The relative abundance of m^6^A sites along *HK2* and *SLC2A1* (GLUT1) mRNA in HCT116 WT and METTL3-KO cells, as detected by m^6^A-seq. The orange rectangles indicated that the m^6^A peaks had a significantly decreased abundance. (k) Agarose electrophoresis and real-time PCR analysis of MeRIP assays in CRC cells showing the direct binding between the m^6^A antibody and *HK2* 5’UTR, *HK2* 3’UTR and *SLC2A1* (GLUT1) 3’UTR. (**l**) MeRIP-qPCR analysis of *HK2* 5’UTR, *HK2* 3’UTR and *SLC2A1* (GLUT1) 3’UTR m^6^A levels in HCT116 WT and METTL3-KO cells, *n* = 3, nonparametric Mann–Whitney test. (WT, wild type; METTL3-KO, METTL3-knockout)

To identify potential mRNA targets of METTL3 whose m^6^A levels were increased by METTL3 in CRC cells, METTL3-knockout and WT HCT116 cells were selected for transcriptome-wide m^6^A-sequencing (m^6^A-seq, MeRIP-seq) and RNA-sequencing (RNA-seq) assays. A noticeable decrease in m^6^A levels was observed in polyadenylated RNAs (poly(A) RNAs) of the METTL3-knockout cells, compared to the WT HCT116 cells, as measured by m^6^A dot blot, EpiQuik™ m^6^A quantification assay and LC-MS/MS analysis (Fig. 4a-c). Consisted with the previous researches, the most common m^6^A motif ‘GGAC’ is significantly enriched in the m^6^A peaks (Fig. 4d). Furthermore, most of the METTL3-binding sites (> 80%) were located in protein-coding transcripts (CDS region) and were highly enriched in 5’UTR and 3’UTR, especially enriched in the vicinity of the stop codon, which was coincidence with the m^6^A distribution (Fig. 4e and Figure S4a).

We next compared the genes with altered-m^6^A modifications between METTL3-knockout cells and the WT cells. The analysis of m^6^A-seq revealed a global hypo-methylation of m^6^A in the transcription level after knockout of METTL3 in HCT116 cells (Fig. 4f-g). A total of 2632 and 584 peaks showed a significant decrease or increase in m^6^A modifications (*p* < 0.05, Table S4) in METTL3-knockout cells relative to WT cells respectively, and thereby they were termed as hypo-methylated and hyper-methylated m^6^A peaks (Figure S4b). Through analysis of the RNA-seq data, we identified 429 hypo-methylated m^6^A genes whose mRNA transcripts were down-regulated (*p* < 0.05, Hypo-down) and 595 hypo-methylated m^6^A genes whose mRNA transcripts were up-regulated (*p* < 0.05, Hypo-up) in METTL3-knockout cells, compared with WT cells (Fig. 4h). Considering the role of METTL3 in the m^6^A methyltransferase complex, mRNA transcripts carrying hypo-methylated m^6^A peaks in HCT116 METTL3 knockout cells were likely potential targets.

We further found that the hypo-down transcripts (*p* < 0.05) were significantly enriched with genes involved in ‘Metabolism of carbohydrates’ and ‘Glucose metabolism’, which included the glycolysis pathway through gene ontology analysis (Figure S4c). Nevertheless, those hypo-up transcripts were significantly enriched with “Post-translational protein modification”, “Chromatin organization”, which are not associated with glycolytic metabolism (Figure S4c). Therefore, the hypo-methylated m^6^A genes, with decreased expression in METTL3-knockout CRC cells (*p* < 0.05) (hypo-down), were selected for further validation (Fig. 4i). Eighteen genes (PYGB, FUT1, GALNS, HK2, IDUA, PC, ENO3, SGSH, ST3GAL2, SLC2A1, HS6ST1, HS3ST1, SLC26A1, NUP62, GLCE, CHST12, ADOGK and AGRN), which involved in ‘Carbohydrates metabolism’ and ‘Glucose metabolism’, were sorted as potential target genes (Fig. 4i). Real-time PCR and MeRIP-qPCR assays were performed to measure the mRNA and m^6^A levels of eighteen hypo-down candidates in different cells. Real-time PCR data showed that the expression of six target genes was significantly impaired both in METTL3-knockout HCT116 (Figure S4d) and METTL3-knockdown SW480 CRC cells (Figure S4e). METTL3 up-regulation increased the RNA level of these six transcripts in DLD1 cells (Figure S4f-g). The m^6^A peaks of these six transcripts were remarkably decreased in METTL3-knockout HCT116 cells, compared with WT cells (Fig. 4j and Figure S4h) according to the MeRIP-seq data. MeRIP-PCR data was consistent with the MeRIP-seq data (Fig. 4k and Figure S4i). Further MeRIP-qPCR [18, 32] analyses showed the m^6^A levels in five of the six target genes were dramatically reduced in HCT116 METTL3-knockout cells (Fig. 4l, Figure S4j). The rest five target genes were further validated by functional glycolysis assay. Lactate production was only significantly reduced in HCT116 cells with *Hexokinase 2* (*HK2*) and *SLC2A1* (GLUT1) downregulation, but not in those cells with the other three genes downregulation (Figure S4k). In addition, we performed MeRIP-seq in 5 CRC patients with METTL3 high expression and 5 CRC patients with METTL3 low expression. The Integrative Genomics View (IGV) data revealed that the m^6^A level of *HK2* and *SLC2A1* (GLUT1) was higher in 5 CRC patients with METTL3 high expression than those with METTL3 low expression (Figure S4l), indicating that higher m^6^A level of *HK2* and *SLC2A1* (GLUT1) depends on METTL3 modification in CRC patients. The MeRIP-seq data in CRC patients is consistent with the data in CRC cell lines. Therefore, we selected *HK2* and *SLC2A1* (GLUT1) as candidate targets of METTL3 for further investigation.

### METTL3 regulates *HK2* and *SLC2A1* (GLUT1) mRNA levels and stability depending on its m^6^A methyltransferase activity

**Fig. 5:**
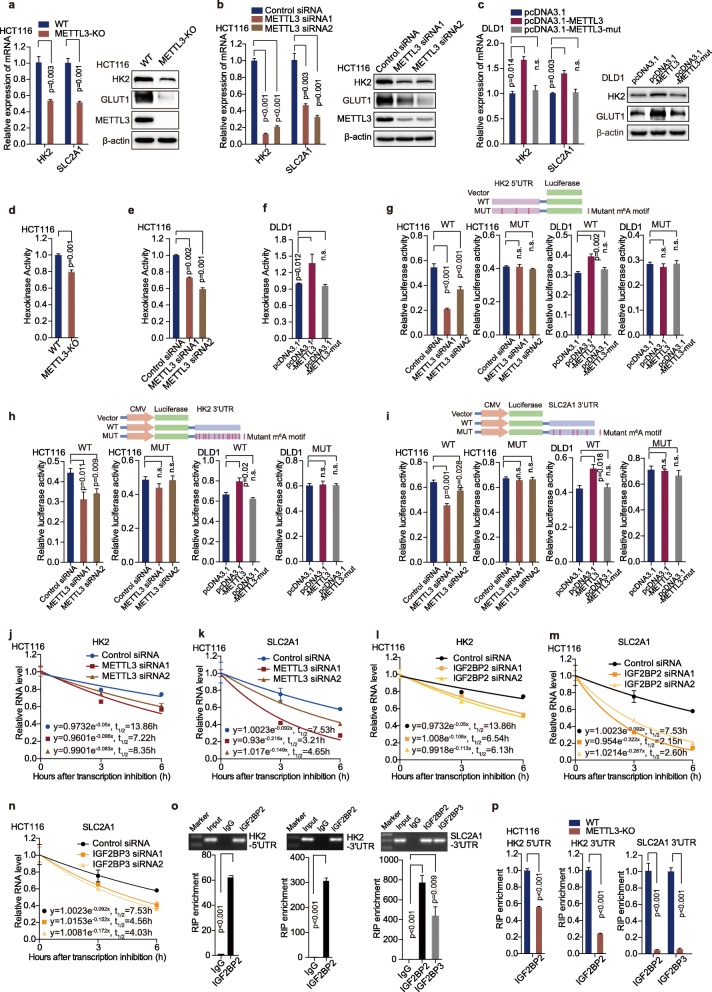
METTL3 regulates *HK2* and *SLC2A1* (GLUT1) mRNA levels and stability depending on its m^6^A methyltransferase activity. (**a-b**) Real-time PCR and Western blot assays were performed to analyze the relative HK2 levels and *SLC2A1* (GLUT1) in HCT116 cells after knockout (**a**) or knockdown (**b**) of METTL3 in HCT116 cells, *n* = 3, nonparametric Mann–Whitney test. (**c**) Real-time PCR and Western blot assays were performed to analyze the relative HK2 levels and *SLC2A1* (GLUT1) in DLD-1 cells after transfection with pcDNA3.1, pcDNA3.1-METTL3 and pcDNA3.1-METTL3-mut, *n* = 3, nonparametric Mann–Whitney test. (**d-e**) Hexokinase activity was measured by colorimetric analysis after knockout (**d**) or knockdown (**e**) of METTL3 in HCT116 cells, *n* = 3, nonparametric Mann–Whitney test. (**f**) Hexokinase activity was measured by colorimetric analysis after transfection with pcDNA3.1, pcDNA3.1-METTL3 and pcDNA3.1-METTL3-mut in DLD1 cells, *n* = 3, nonparametric Mann–Whitney test. (**g-i**) Luciferase activity was measured in HCT116 cells transfected with control siRNA, METTL3 siRNA, pcDNA3.1, pcDNA3.1-METTL3 and pcDNA3.1-METTL3-mut. The luciferase reporters expressing WT or mutant human *HK2* 5’UTRs (**g**) and WT or mutant human *HK2* 3’UTRs (**h**) and WT or mutant human *SLC2A1* (GLUT1) 3’UTRs (**i**) were used. The results were showed in the form of relative firefly luciferase activity normalized to Renilla luciferase activity. *n* = 4, nonparametric Mann–Whitney test. (**j-k**) The *HK2* and *SLC2A1* (GLUT1) mRNA half-life (t_1/2_) was detected by real-time PCR in HCT116 cells transfected with control siRNA or METTL3 siRNA1/2, *n* = 3, nonparametric Mann–Whitney test. (**l**) The HK2 mRNA half-life (t_1/2_) was detected by real-time PCR in HCT116 cells transfected with control siRNA or IGF2BP2 siRNA1/2, *n* = 3, nonparametric Mann–Whitney test. (**m-n**) The *SLC2A1* (GLUT1) mRNA half-life (t_1/2_) was detected by real-time PCR in HCT116 cells transfected with control siRNA, IGF2BP2 or IGF2BP3 siRNA1/2, *n* = 3, nonparametric Mann–Whitney test. (**o**) Agarose electrophoresis and real-time PCR analysis of RIP assays in CRC cells showing the direct binding between the IGF2BP2 protein with HK2 5’UTR and HK2 3’UTR, and as well as the direct binding between IGF2BP2/3 with SLC2A1 (GLUT1) 3’UTR. (**p**) RIP-qPCR revealed the binding enrichment of IGF2BP2 to HK2 5’UTR and HK2 3’UTR, as well as the binding enrichment of IGF2BP2/3 to SLC2A1 (GLUT1) HK2 3’UTR, were decreased following knockout of METTL3 in HCT116 cells, *n* = 3, nonparametric Mann–Whitney test

In biological validation assay, *HK2* and *SLC2A1* (GLUT1) expression were confirmed to be downregulated in METTL3-knockout cells (Fig. 5a) and METTL3-knockdown cells (Fig. 5b and Figure S5a). In addition, overexpression of METTL3, but not METTL3-mut dramatically increased *HK2* and *SLC2A1* (GLUT1) mRNA and protein levels (Fig. 5c). We further measured m^6^A levels of glycolysis genes *HK2* and *SLC2A1* (GLUT1) by MeRIP-qPCR after transfection of control, METTL3 and METTL3-mut plasmids in DLD1 cells. The MeRIP-qPCR data showed that overexpression of METTL3, but not METTL3-mut, dramatically increased the m^6^A levels of *HK2* and *SLC2A1* (GLUT1) in DLD1 cells (Figure S5b). It indicates that m^6^A modification of *HK2* and *SLC2A1* (GLUT1) directly affects its mRNA level. We detected the hexokinase activity in CRC cells as well. The hexokinase activity was noticeably decreased in METTL3-knockout cells and METTL3-knockdown cells (Fig. 5d-e and Figure S5c). Moreover, overexpression of METTL3, but not METTL3-mut, significantly up-regulated the hexokinase activity (Fig. 5f). To further address the effect of m^6^A modification on *HK2* and *SLC2A1* (GLUT1) gene, we constructed both wild-type and mutant *HK2* and *SLC2A1* (GLUT1) luciferase reporter plasmids, which contained the wild-type 5′ and 3’UTR of *HK2*, the 3’UTR of *SLC2A1* (GLUT1) or mutations (m^6^A was replaced by T) in the m^6^A sites (Fig. 5g-i). As expected, compared with control siRNA, METTL3 siRNA substantially reduced luciferase activity of the individual reporter constructs bearing wild-type *HK2* 5′ and 3’UTR that have intact m^6^A sites, and wild-type 3’UTR of *SLC2A1* (GLUT1). However, downregulation of METTL3 had no significant effect on the luciferase activity in these reporter plasmids with m^6^A sites mutations in HCT116 (Fig. 5g-i) and SW480 cells (Figure S5d-f). Furthermore, overexpression of METTL3, but not METTL3 mutant, significantly increased luciferase activity of the individual reporter constructs bearing wild-type *HK2* 5′ and 3’UTR, and wild-type *SLC2A1* (GLUT1) 3’UTR in DLD1 cells (Fig. 5g-i). Overexpression of METTL3 and METTL3-mut had no significant effect on *HK2* and *SLC2A1* (GLUT1) luciferase reporter plasmids with m^6^A sites mutation (Fig. 5 g-i).

To analyze the effect of m^6^A modification on the stability of METTL3 target transcripts, we conducted RNA stability assays [8]. The RNA stability curves showed that knockdown of METTL3 reduced the half-life of *HK2* and *SLC2A1* (GLUT1) mRNA in HCT116 (Fig. 5j-k) and SW480 cells (Figure S5g-h). Thus, METTL3-induced upregulation of *HK2* and *SLC2A1* (GLUT1) level is at least in part due to the increased stability of *HK2 and SLC2A1* mRNA transcript. Insulin-like growth factor 2 mRNA binding proteins (IGF2BP1, IGF2BP2 and IGF2BP3) are three well-established m^6^A readers in mammalian cells [33], and responsible for stabilizing mRNA. m^6^A readers YTHDF2, YTHDF3 and YTHDC2 are responsible for mRNA decay [34–36]. Real-time PCR data showed that knockdown of IGF2BP2, but not IGF2BP1/3 significantly reduced the mRNA level of *HK2* in HCT116 and SW480 cells (Figure S5i). Downregulation of IGF2BP2/3, but not IGF2BP1 noticeably reduced the mRNA level of *SLC2A1* (GLUT1) (Figure S5j). Knockdown of YTHDF2, YTHDF3 or YTHDC2 did not influence the mRNA levels of *HK2* or *SLC2A1* (GLUT1) (Figure S5k-l). Furthermore, the half-life of *HK2* mRNA was significantly decreased in HCT116 cells after transfection with IGF2BP2 siRNA (Fig. 5l), and the half-life of *SLC2A1* (GLUT1) mRNA was significantly reduced in IGF2BP2/3 down-regulated HCT116 cells (Fig. 5m-n). RIP assay showed that IGF2BP2 directly bound to the 5′ and 3’UTR of *HK2* mRNA (Fig. 5o) and IGF2BP2/3 directly bound to the 3’UTR of *SLC2A1* (GLUT1) mRNA (Fig. 5o). Further RIP real-time PCR assay indicated that knockout of METTL3 significantly reduced the binding efficiency of IGF2BP2 to the 5′/3’UTR of *HK2* mRNA and the binding efficiency of IGF2BP2/3 to the 3’UTR of *SLC2A1* (GLUT1) mRNA (Fig. 5p). Taken together, our data demonstrate that METTL3-mediated m^6^A modification increases *HK2* and *SLC2A1* (GLUT1) expression through IGF2BP2-dependent and IGF2BP2/3-dependent mRNA stability regulation, respectively.

### *HK2* and *SLC2A1* (GLUT1) are functionally essential target genes of METTL3 in CRC

**Fig. 6:**
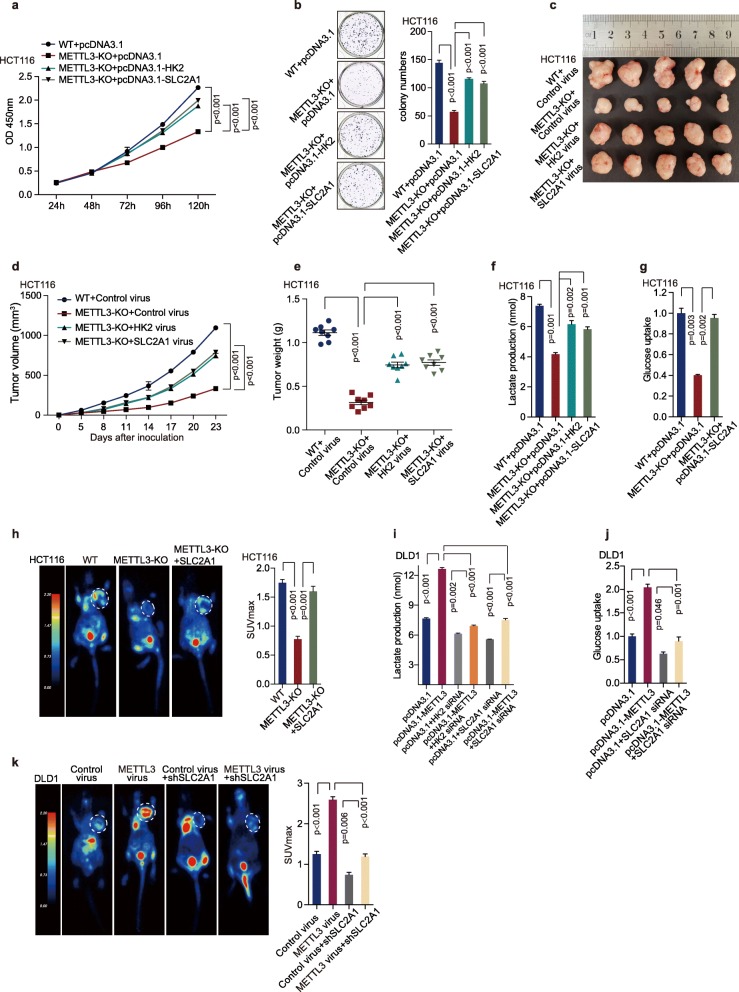
*HK2* and *SLC2A1* were functionally important target genes of METTL3 in CRC. (**a-b**) CCK8 assays (**a**) and Colony formation assay (**b**) were measured after transfection with pcDNA3.1, pcDNA3.1-*HK2* and pcDNA3.1-*SLC2A1* plasmid in HCT116 WT and METTL3-knockout cells. Nonparametric Mann–Whitney test. (**c-e**) Representative images of tumors (**c**), statistical analysis of tumor volumes (**d**) and tumor weights (**e**) in nude mice bearing HCT116 cells in different groups (*n* = 8, nonparametric Mann–Whitney test). (**f**) Lactate production assay was performed after transfection with pcDNA3.1, pcDNA3.1-*HK2* and pcDNA3.1-*SLC2A1* plasmid in HCT116 WT and METTL3-KO cells, *n* = 3, Nonparametric Mann–Whitney test. (**g**) Glucose uptake was measured after transfected with pcDNA3.1, and pcDNA3.1-*SLC2A1* plasmid in HCT116 WT and METTL3-knockout cells, *n* = 3, Nonparametric Mann–Whitney test. (**h**) Representative images of ^18^F-FDG uptake by micro-PET imaging in nude mice bearing HCT116 cells in different groups. White circles indicated tumor glucose uptake. Maximum uptake values (SUV_max_) for xenografts measured by FDG-PET were presented; nonparametric Mann–Whitney test. (**i**) Lactate production assay was performed in DLD1 cells with different treatment, *n* = 3, nonparametric Mann–Whitney test. (**j**) Glucose uptake was detected in DLD1 cells with different treatment, *n* = 3, nonparametric Mann–Whitney test. (**k**) Representative images of ^18^F-FDG uptake by micro-PET imaging in nude mice bearing DLD1 cells in different groups. White circles indicated tumor glucose uptake. Maximum uptake values (SUV_max_) for xenografts measured by FDG-PET were presented; nonparametric Mann–Whitney test. Fig. 2k and Fig. 6k shared experimental controls and METLL3-overexpression group. (WT, wild type; METTL3-KO, METTL3-knockout)

Hexokinase 2 (HK2) is a pivotal kinase in the glycolytic pathway [37]. Previous studies have demonstrated that HK2 activity is remarkably increased in various malignant neoplasms, as well as in CRC [38, 39]. Glucose transporter 1 (GLUT1), encoded by *SLC2A1*, is the predominant glucose transporter expressed on colonic epithelial cells [40]. Here we first revealed that METTL3 increased *HK2* and *SLC2A1* (GLUT1) expression via promoting their mRNA stability. Therefore, we next performed rescue experiments to investigate whether *HK2* and *SLC2A1* (GLUT1) participated in the biological function of METTL3 in CRC. HCT116 WT and METTL3-knockout cells were transfected with control, *HK2* or *SLC2A1* overexpression plasmids. The overexpression efficiency of *HK2* and *SLC2A1* were confirmed by real-time PCR and Western blot analysis (Figure S6a-b). Ectopic expression of *HK2* or *SLC2A1* partially restored the proliferation (Fig. 6a) and colony formation ability (Fig. 6b) of METTL3-knockout cells and tumor growth (Fig. 6c-e, Figure S6c-e). In addition, DLD1 cells were transfected with METTL3 overexpression plasmid and then treated with *HK2* or *SLC2A1* siRNA. The downregulation efficiency of HK2 and SLC2A1 siRNA were confirmed by real-time PCR and Western blot analysis (Figure S6f-g). Downregulation of *HK2* or *SLC2A1* dramatically impaired METTL3-induced cell proliferation (Figure S6h) and colony formation (Figure S6i) in vitro and tumor growth in vivo (Figure S6j-l), which further supported *HK2* and *SLC2A1* (GLUT1) as critical target genes of METTL3 in CRC.

In glycolytic assays, ectopic expression of *HK2* or *SLC2A1* (GLUT1) restored the decrease of lactate production in HCT116 METTL3-knockout cells (Fig. 6f). Meanwhile, overexpression of *SLC2A1* (GLUT1) significantly restored the decrease of glucose uptake in HCT116 METTL3-knockout cells in vitro (Fig. 6g) and in vivo (Fig. 6h). Furthermore, the downregulation of *HK2* or *SLC2A1* (GLUT1) significantly reduced METTL3-mediated higher lactate production in DLD1 cells (Fig. 6i). Downregulation of *SLC2A1* (GLUT1) dramatically impaired METTL3-induced higher glucose uptake in DLD1 cells in vitro (Fig. 6j) and in vivo (Fig. 6k). Thus, *HK2* and *SLC2A1* (GLUT1) mediate the regulatory function of METLL3 in CRC cells.

### The levels of METTL3 and glycolysis components correlate and are clinically relevant in CRC patients

**Fig. 7:**
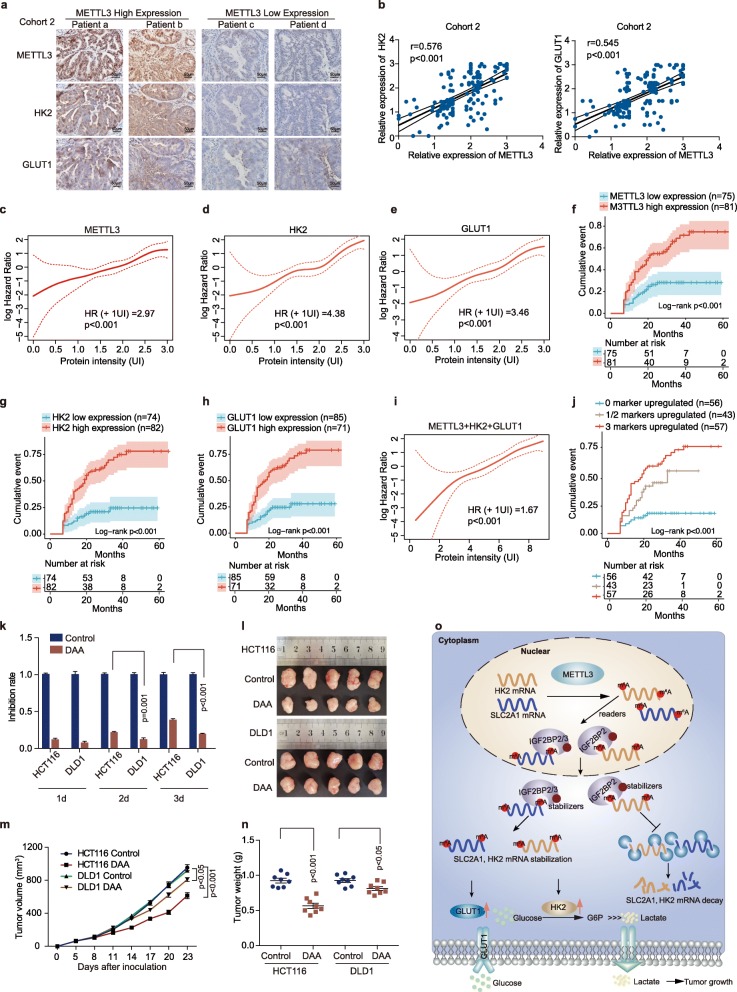
The levels of METTL3 and glycolysis components correlate and are clinically relevant in CRC patients. (**a**) Representative immunohistochemical images of METTL3, HK2 and GLUT1 in CRC tissues using IHC analysis in Cohort 2. Scale bars: 50 μm (400X). Fig. 7a and Figure S7a shared representative immunohistochemical images of METTL3 in METTL3 high expression and METTL3 low expression groups. (**b**) Correlation between METTL3 expression and HK2 IHC scores in CRC tissues of Cohort 2 (left). Correlation between METTL3 expression and GLUT1 IHC scores in CRC tissues of Cohort 2 (Right), *n* = 156. (**c-e**) Smooth estimates of HR (+ 1 UI; increase in recurrence risk for every unit of staining intensity) showed a higher risk of relapse for patients with higher expression of METTL3 (**c**), HK2 (**d**) or GLUT1 (**e**) in Cohort 2. Red dashed lines Indicated 95% confidence interval. (**f-h**) Kaplan-Meier curves showing the disease-free survival of patients with different levels of METTL3 (**f**), HK2 (**g**) or GLUT1 (**h**) protein intensity in Cohort 2. (**i**) Smooth estimates of HR (+ 1 UI) showed a higher risk of relapse for patients with higher combined scores of molecular markers (METTL3 + HK2 + GLUT1) in Cohort 2. Red dashed lines Indicated 95% confidence interval. (**j**) Kaplan-Meier analysis of disease-free survival for CRC patients based on the number of upregulated molecular markers (METTL3, HK2 and GLUT1) in Cohort 2. (**k**) Inhibition rate of DAA (3-Deazaadenosine) (100 μM) in HCT116 and DLD1 cells. (**l-n**) Representative images of tumors (**l**), statistical analysis of tumor volume (**m**), and weights (**n**) in nude mice bearing HCT116 and DLD1 cells treated with DAA. (**o**) Schematic diagram of the relationship among METTL3, m^6^A modification, colorectal cancer cell progression and glycolysis metabolism

We next performed immunohistochemical staining in CRC patients’ tissues of Cohort 2. Interestingly, the samples with METTL3 higher expression displayed strongly staining for HK2 and GLUT1 (Fig. 7a, left panel). In addition, samples with low expression of METTL3 appeared low levels of HK2 and GLUT1 (Fig. 7a, right panel). Statistically, METTL3 expression was positively correlated with HK2 and GLUT1 expression in CRC tissues (Fig. 7b). There was no significant correlation between METTL3 and IGF2BP2/3 expression in CRC tissues of Cohort 2 (Fig. S7a). We next assessed the association between the intensity of METTL3, glycolysis components, and disease-free survival after tumor resection in this patients’ cohort. This analysis showed that elevated expression of METTL3, HK2 or GLUT1 in CRC tissues predicted robustly shorter disease-free intervals, either as a linear (Fig. 7c-e) or categorized variable (Fig. 7f-h). Furthermore, a linear association was found between the combined expression of METTL3, HK2 and GLUT1 and the risk of relapse after therapy in CRC (Fig. 7i). The shortest disease-free survival times were detected in those patients with three highly expressed markers METTL3/HK2/GLUT1 (Fig. 7j). From these observations, we conclude that elevated expression levels of METTL3 and its target genes may identify CRC patients with poor prognosis. We next analyzed the correlation of METTL3, HK2 or GLUT1 expression and different clinicopathological features in Cohort 2. METTL3 expression was positively correlated with pathological differentiation, AJCC stage, and recurrence in CRC patients (Figure S7b). HK2 and GLUT1 expression were positively correlated with AJCC stage, and recurrence (Figure S7b). In addition, METTL3 expression is gradually increased from normal colorectal epithelial tissues, adenoma to cancer tissues in Cohort 3 (Figure S7c).

So far, we have proved that METTL3 may function as an oncogene by stabilizing *HK2* and *SLC2A1* mRNA levels in CRC cells. Recently, several RNA methyltransferase inhibitors have been developed as anti-tumor drug candidates. We next hypothesized that those CRC patients, harboring higher METTL3 expression, will be more sensitive to the anti-tumor drug candidates targeting METTL3. To test this prediction, we treated the HCT116 CRC cells, which express a higher METTL3 level, with DAA (3-Deazaadenosine, an inhibitor of the internal N^6^-Methyladenosine) [41, 42]. DAA treatment resulted in 39% inhibition of cell proliferation in HCT116 cells. Conversely, treatment in DLD1, which has a lower expression of METTL3, with the same dose of DAA, resulted in only 20% inhibition of cellular proliferation (Fig. 7k). These inhibition results could also be recapitulated in vivo. DAA is more efficient to inhibit tumor growth (Fig. 7l-m) and tumor weight (Fig. 7n) in nude mice bearing HCT116 cells than those bearing DLD1 cells. Taken together, these data suggest that targeting the METTL3 may be more effective in CRC cells with higher METTL3 expression than in those CRC cells with lower METTL3 expression.

## Discussion

Various epigenetic modifications, including DNA methylation, histone modification, microRNA, and long non-coding RNA regulation, may contribute to colorectal carcinogenesis [43–45]. However, the potential involvement of RNA m^6^A modification is poorly defined in human colorectal cancer (CRC) and glycolytic metabolism. Through a combination of genomic, biochemical, and cell biological analyses, we have demonstrated that m^6^A RNA methylation levels and RNA methyltransferase METTL3 expression are highly increased in CRC patients with higher FDG uptake. GSEA analyses have demonstrated that cell proliferation and glycolytic pathways in cancer are significantly enriched in response to METTL3 alteration in the CRC cells. The bioinformatics analyses have been functionally validated in several in vitro and in vivo experimental models. In cultured CRC cells and xenograft mouse models, downregulation of METTL3 markedly suppresses tumor growth and inhibits glycolysis progression in CRC. The data consistently point to the notion that higher METTL3 expression and higher m^6^A methylation level are decisive factors of controlling human CRC aggressiveness.

RNA Methyltransferase METTL3 may participate in regulating the mRNA stability and modification of target genes [46]. However, the underlying molecular mechanisms of METTL3-regulated genes in CRC remain unknown. Our transcriptome-wide m^6^A-seq assay, the subsequent validation and functional studies suggest that *HK2* and *SLC2A1* (GLUT1) are the critical target genes of METTL3 in CRC. As an m^6^A RNA methyltransferase, METTL3 increases the m^6^A level of *HK2* gene mainly at 3′ and 5′ untranslated regions (UTRs) and the m^6^A level of SLC2A1 gene mainly at 3′UTR, which in turn leads to the up-regulation of HK2 and *SLC2A1* (GLUT1) at the RNA level and protein level. In addition, our luciferase reporter/mutagenesis assays indicate that the m^6^A sites in the UTRs of METTL3 critical target genes such as *HK2* and *SLC2A1* are essential for METTL3 to post-transcriptionally regulate their expression. This finding was further supported by the MeRIP assay, RNA stabilization assay, and qRT-PCR in control and METTL3-knockout or METTL3-knockdown CRC cells. Our data and previous studies [38, 39] demonstrate that *HK2* and *SLC2A1* may act as oncogenes to promote cell glycolysis metabolism. Here, we also firstly reveal that HK2 and GLUT1 participate in METTL3-mediated biological function in CRC. Thus, the METTL3➔HK2/GLUT1 axis likely plays a critical role in the pathogenesis of CRC. A schematic model summarizing our discoveries is shown in Fig. 7o.

Previous studies suggest that mRNA transcripts with m^6^A modifications tend to be regulated by YTHDFs or IGF2BPs as the direct m^6^A readers [8–10, 47]. Primarily due to the recognition by different m^6^A readers, the mRNA transcripts with m^6^A modifications have a different fate. YTHDF2, YTHDF3, and YTHDC2 tend to reduce the expression of genes by promoting their mRNA decay [34–36], while IGF2BPs tend to regulate the gene expression by promoting mRNA stability [33]. We showed that depleted expression of METTL3 substantially shortened the half-life of its critical targets such as *HK2* and *SLC2A1*, suggesting that METTL3-mediated increase in *HK2* and *SLC2A1* expression are at least in part due to the increased stability of these two mRNA transcripts. In the further validation study, we demonstrated that METTL3 epigenetically stabilized *HK2* and *SLC2A1* mRNA levels through an m^6^A-IGF2BP2 and m^6^A-IGF2BP2/3-dependent mechanism, and therefore support the malignant state of CRC cells. Since 1) the mRNA levels of *HK2* and *SLC2A1* were significantly reduced after genetic deficiency of IGF2BP2 or IGF2BP2/3; 2) IGF2BP2 directly bound with the 5’UTR/3’UTR of *HK2* mRNA, and IGF2BP2/3 directly bound with the 3’UTR of *SLC2A1* mRNA; 3) Knockdown of METTL3 significantly disrupted the binding of IGF2BP2 to the 5’UTR/3’UTR of *HK2* mRNA, and the binding of IGF2BP2/3 to the 3’UTR regions of *SLC2A1* mRNA. In support of our observation, IGF2BP2 exhibits oncogenic function as m^6^A readers, which associated with m^6^A reading processes in tumorigenesis and progression [33, 48]. Dysregulation of IGF2BPs could result in abnormal accumulation of oncogenic products such as MYC in human cancer cells [33].

Therefore, from a therapeutic perspective, the mechanistic understanding of METTL3-induced cancer cell biological function and glucose metabolism in cellular regulation will enable the identification of the novel therapeutic targets. Profiling of m^6^A modification pattern that is affected by RNA methyltransferase may also allow the development of diagnostic tests of cancer. In short, METTL3 plays an oncogenic role in stabilizing *HK2* and *SLC2A1* mRNA via IGF2BPs, and further regulate glycolytic metabolism as well as cell proliferation in CRC cells. In addition to METTL3 biological and epigenetic importance, our work may be relevant to the clinical management of CRC patients. As the higher expressions of METTL3, HK2, and GLUT1 are associated with poor outcomes in CRC patients, METTL3 and its target genes may be promising biomarkers to guide early diagnosis and therapy in CRC. We further found that DAA (a chemical inhibitor of the internal N^6^-Methyladenosine) is more effective to inhibit cell proliferation in CRC cells with higher METTL3 expression than those cells with lower METTL3 expression. Since DAA inhibits other signal pathways [42, 49], a more specific inhibitor of METTL3 or glycolysis pathway needs to be developed for CRC patients, especially for those CRC patients with higher METTL3 expression treatment. Taken together, METTL3 and its associated pathway are crucial for colorectal carcinogenesis as well as glycolysis pathway, and targeting this pathway may be pivotal in the prevention and treatment of colorectal cancer.

## Conclusions

Here, for the first time, we found that m^6^A modification is closely correlated with glycolysis pathway activation in colorectal cancer patients’ tissues. Mechanically, HK2 and GLUT1 were found to be regulated by m^6^A modification and participate in glycolysis activation in colorectal cancer. The METTL3➔HK2/GLUT1-IGF2BPs axis likely plays a critical role in the pathogenesis of colorectal cancer. Targeting METTL3 and its pathway may be promising for treating colorectal cancer patients with high glucose metabolism.

## Supplementary information

**Additional file 1.** Supplementary Methods

**Additional file 2 Table S1**. RNA-seq analysis in HCT116 WT and METTL3-knockout cells.

**Additional file 3 Figure S1**. METTL3 is closely correlated with glycolytic metabolism in CRC. (**a**) The relative expression of METTL3 was measured by real-time PCR in normal colonic epithelial cell line FHC and CRC cell lines, *n* = 3, nonparametric Mann–Whitney test. (**b**) Schematic diagram of METTL3 genomic sequence in HCT116 wild-type (WT) and METTL3-knockout (METTL3-KO) cells. Red label, target sgRNA sequence; Yellow label, A 161 bp DNA fragment was inserted in the cutting site; Red circle, a premature stop site was generated after insertion of 161 bp DNA fragment. (**c**) The knockout efficiency of METTL3 was confirmed by Western blot. (**d**) GSEA analysis was conducted to identify the differential gene profiles between HCT116 METTL3-KO and WT cells. (WT, wild type; METTL3-KO, METTL3-knockout). **Figure S2**. METTL3 drives glycolytic metabolism in CRC. (**a-b**) The knockdown efficiency of METTL3 siRNA1/2 was evaluated in HCT116 (**a**) and SW480 (**b**) cells, *n* = 3, nonparametric Mann–Whitney test. (**c-e**) Lactate production (**c**), glucose uptake (**d**) and ECAR (**e**) were measured after transfection of control siRNA and METTL3 siRNA1/2 in SW480 cells, *n* = 3, nonparametric Mann–Whitney test. (**f**) OCR was measured in HCT116 WT and METTL3-KO cells (left). OCR was measured in SW480 cells transfected with control siRNA and METTL3 siRNA1/2 (right). (**g**) Schematic domain structures of METTL3. METTL3 MTase (AA residues 369–580). (**h-i**) Real-time PCR (**h**) and Western blot assay (**i**) were performed to detect METTL3 expression after transfection with pcDNA3.1-METTL3 and pcDNA3.1-METTL3-mut in DLD1 cells. (WT, wild type; METTL3-KO, METTL3-knockout). **Figure S3**. METTL3 is an oncogenic gene in colorectal cancer. (**a-b**) GSEA analysis was conducted to identify the differential gene profiles between HCT116 METTL3-KO and WT cells. (**c**) The expression of CDK1, PCNA and CDCA7 were detected in HCT116 WT and METTL3-KO cells, *n* = 3, nonparametric Mann–Whitney test. (**d-e**) The expression of CDK1, PCNA and CDCA7 were detected after transfection with control siRNA and METTL3-siRNA1/2 in HCT116 (**d**) and SW480 cells (**e**), *n* = 3, nonparametric Mann–Whitney test. (**f**) Cell proliferation of HCT116 cells was measured by CCK8 assay after transfected with control siRNA or METTL3 siRNA, *n* = 6, nonparametric Mann–Whitney test. (**g**) Colony formation assay was performed in HCT116 cells after transfection with control siRNA or METTL3 siRNA1/2, *n* = 3, nonparametric Mann–Whitney test. (**h**) Cell proliferation assay was performed by CCK8 assay in SW480 cells with METTL3 knockdown, *n* = 6, nonparametric Mann–Whitney test. (**i**) Colony formation assay was performed after transfection with control siRNA or METTL3 siRNA1/2 in SW480 cells, *n* = 3, nonparametric Mann–Whitney test. (**j**) Representative images of tumors in nude mice bearing DLD1 cells treated with PBS, control adenovirus and shControl adenovirus, *n* = 8. (**k-l**) Tumor volumes and (**k**) tumor weights (**l**) were measured in mice bearing DLD1 cells treated with PBS, control adenovirus and shControl adenovirus, *n* = 8, nonparametric Mann–Whitney test. (WT, wild type; METTL3-KO, METTL3-knockout). **Figure S4**. Transcriptome-wide m6A-seq and RNA-seq assays identified potential targets of METTL3 in colorectal cancer. (**a**) The distribution of m6A IP signal across the mRNA transcripts. m6A IP signal was enriched around the stop codon of mRNAs. (**b**) Comparison of the abundance of m6A peaks between HCT116 WT and METTL3-KO cells. A total of 2632 (hypo-methylated) and 584 (hyper-methylated) m6A peaks showed a significant decrease and increase in abundance in HCT116 METTL3-KO cells compared with WT cells. (**c**) Gene ontology analysis of genes with a significant decrease in m6A modification as well as a significant decrease in mRNA expression level (Hypo-down, *p* < 0.05) in HCT116 METTL3-KO cells (up). Gene ontology analysis of genes with a significant decrease in m6A modification as well as a significant increase in mRNA expression level (Hypo-up, *p* < 0.05) in HCT116 METTL3-KO cells (down). (d-e) Relative mRNA expression of METTL3 downstream targets from ‘Metabolism of carbohydrates pathway’ and ‘Glucose metabolism’ of GO analysis in HCT116 WT, HCT116 METTL3-KO (**d**) and SW480 cells (**e**) treated with control siRNA or METTL3 siRNA transfection, *n* = 3, nonparametric Mann–Whitney test. (**f**) Relative mRNA expression of METTL3 downstream targets in DLD1 cells transfected with METTL3 overexpression plasmids, *n* = 3, nonparametric Mann–Whitney test. (**g**) Venn diagram showed 6 genes with consistently decreased levels in both HCT116 and SW480 cells with METTL3 knockout or knockdown and increased expression in DLD1 cells with METTL3 upregulated. (**h**) The relative abundance of m6A sites along SLC26A1, PC, GLCE and CHST12 mRNA in HCT116 WT and METTL3-KO cells, as detected by m6A-seq. (**i**) Agarose electrophoresis and MeRIP-qPCR assays in CRC cells showing the direct binding between the m6A antibody and SLC26A1, PC, GLCE and CHST12. (**j**) MeRIP-qPCR analysis of SLC26A1, PC, GLCE and CHST12 RNA m6A levels in HCT116 cells with METTL3 knockout, *n* = 3, nonparametric Mann–Whitney test. (**k**) Lactate production was detected in HCT116 cells after knockdown of HK2, SLC2A1, PC, GLCE and CHST12, respectively, *n* = 3, nonparametric Mann–Whitney test. (**l**) The relative abundance of m6A sites along HK2 and SLC2A1 (GLUT1) mRNA in CRC tissues with high or low METTL3 expression as detected by m6A-seq. The orange rectangles indicated that the m6A peaks had a significant decreased abundance. (WT, wild type; METTL3-KO, METTL3-knockout). **Figure S5**. METTL3 regulates HK2 and SLC2A1 (GLUT1) mRNA levels and stability depending on its m6A methyltransferase activity. (**a**) Real-time PCR and Western blot assay were performed to detect the levels of HK2 and SLC2A1 (GLUT1) in SW480 cells after transfection of control siRNA and METTL3 siRNA1/2, *n* = 3, nonparametric Mann–Whitney test. (**b**) MeRIP-qPCR analysis of HK2 and SLC2A1 m6A levels in DLD1 cells transfected with control, METTL3 overexpression and METTL3-mut plasmids, *n* = 3, nonparametric Mann–Whitney test. (**c**) Hexokinase activity was measured in SW480 cells transfected with METTL3 siRNA1/2, *n* = 3, nonparametric Mann–Whitney test. (**d**) Relative luciferase activity of WT or mutant HK2 5’UTR firefly luciferase reporter in SW480 cells treated with control siRNA or METTL3 siRNA1/2, *n* = 4, nonparametric Mann–Whitney test. (**e**) Relative luciferase activity of WT or mutant HK2 3’UTR firefly luciferase reporter in SW480 cells treated with control siRNA or METTL3 siRNA1/2, *n* = 4, nonparametric Mann–Whitney test. (**f**) Relative luciferase activity of WT or mutant SLC2A1 (GLUT1) 3’UTR firefly luciferase reporter in SW480 cells treated with control siRNA or METTL3 siRNA1/2, *n* = 4, nonparametric Mann–Whitney test. (**g-h**) The HK2 (**g**) and SLC2A1 (GLUT1) (**h**) mRNA half-life (t1/2) were detected by real-time PCR in SW480 cells transfected with control siRNA or METTL3 siRNA1/2, *n* = 3, nonparametric Mann–Whitney test. (**i**) The relative expression of HK2 mRNA was measured by real-time PCR in HCT116 and SW480 cells after transfection with control, IGF2BP1, IGF2BP2 or IGF2BP3 siRNA, respectively, *n* = 3, nonparametric Mann–Whitney test. (**j**) The relative expression of SLC2A1 (GLUT1) mRNA was measured by real-time PCR in HCT116 and SW480 cells after transfection with control, IGF2BP1, IGF2BP2 or IGF2BP3 siRNA, respectively, *n* = 3, nonparametric Mann–Whitney test. (**k-l**) The relative expression of HK2 (**k**) and SLC2A1 (GLUT1) (**l**) mRNA were measured by real-time PCR in HCT116 cells or SW480 cells after knockdown of YTHDF2, YTHDF3 or YTHDC2, respectively, *n* = 3, nonparametric Mann–Whitney test. **Figure S6**. HK2 and SLC2A1 (GLUT1) are functionally important target genes of METTL3 in CRC (**a**) The overexpression efficiency of HK2 was confirmed by real-time PCR and Western blot analysis. (**b**) The overexpression efficiency of GLUT1 was confirmed by real-time PCR and Western blot analysis. (**c-e**) Representative images (**c**), tumor volumes (**d**) and tumor weights (**e**) in nude mice bearing HCT116 WT cells treated with PBS and control adenovirus, *n* = 8, nonparametric Mann–Whitney test. (**f**) The downregulation efficiency of HK2 siRNA was confirmed by real-time PCR and Western blot analysis. (**g**) The downregulation efficiency of GLUT1 siRNA was confirmed by real-time PCR and Western blot analysis. (**h-i**) Cell proliferation assay (**h**) and colony formation assay (**i**) were performed in DLD1 cells with different treatment, *n* = 6, nonparametric Mann–Whitney test. (**j-l**) Representative images of tumors (**j**), statistical analysis of tumor volumes (**k**) and tumor weights (**l**) in nude mice bearing DLD1 cells in different groups (*n* = 8, nonparametric Mann–Whitney test). Fig. 3h, Fig. 3m and Figure S6j shared experimental controls and METLL3 overexpression group. **FigureS7**. METTL3 is not correlated with IGF2BP2/3. (**a**) Representative immunohistochemical images of METTL3, IGF2BP2 and IGF2BP3 in CRC tissues using IHC analysis in Cohort 2. Fig. 7a and Figure S7a shared representative immunohistochemical images of METTL3 in METTL3 high expression and METTL3 low expression groups. (**b**) Comparing age, gender, histological differentiation, AJCC stage and recurrence between METTL3/HK2/GLUT1 high and low expression tumors of Cohort 2. The heat map illustrated the association of different clinicopathological features with METTL3/HK2/ GLUT1 high and low expression, Chi-square test. (**c**) Statistical analysis of METTL3 expression in human colorectal cancer (*n* = 67), colorectal adenoma (*n* = 75) and normal colorectal tissues (*n* = 126) of Cohort 3.)

**Additional file 4 Table S2**. Pathways regulated by METTL3 in HCT116 cells.

**Additional file 5 Table S3**. Metabolites of glycolysis pathway measured by Liquid chromatography-coupled tandem mass spectrometry (LC-MS/MS) in HCT116 WT and METTL3-knockout cells.

**Additional file 6 Table S4**. m6A-seq analysis in HCT116 WT and METTL3-knockout cells.

**Additional file 7 Table S5**. Clinical information of 47 cases CRC patients in Cohort 1.

**Additional file 8 Table S6**. Clinical information of 156 cases CRC patients in Cohort 2.

**Additional file 9 Table S7**. Clinical information of 30 cases CRC patients in GSE110225.

**Additional file 10 Table S8**. The sequences of siRNAs and primers used this study.

## Data Availability

The raw sequencing data have been deposited in the Gene Expression Omnibus database under the accession number GSE130012. All the other data generated in this study are included in the article and the additional files.

## Abbreviations

m^6^A: N6-methyladenosine
CRC: Colorectal cancer
^18^F-FDG PET: [^18^F]-Fluoro-2-deoxyglucose positron emission tomography
SUV_max_: Standardized uptake value
LC-MS/MS: Liquid chromatography-tandem mass spectrometry
ECAR: Extracellular acidification rate
OCR: Oxygen consumption rate
METTL3: Methyltransferase-like 3
GLUT1: Glucose transporter 1
HK2: Hexokinase 2
MeRIP: Methylated RNA immunoprecipitation
RIP: RNA Immunoprecipitation
DAA: 3-Deazaadenosine
