# Supplementary material for: m6A-dependent glycolysis enhances colorectal cancer progression
Source: Mol Cancer. 2020 Apr 3;19:72. doi: 10.1186/s12943-020-01190-w (PMC7118901; doi:10.1186/s12943-020-01190-w)
Supplement: Supplementary file 1 — Additional file 1. Supplementary Methods [file 12943_2020_1190_MOESM1_ESM.docx]

**Supplementary Methods**

**Patient specimens**

We used three Cohorts of patients with colorectal cancer who underwent surgery between 2012 and 2019. Cohort 1 (fresh tissues and paraffin-embedded tissues) were from Xuzhou Central Hospital, Xuzhou Medical University; Cohort 2 (paraffin-embedded tissues) were from the Eastern Campus of Renji Hospital, Shanghai Jiao Tong University School of Medicine; Cohort 3 (fresh tissues) were from the Western Campus of Renji Hospital, Shanghai Jiao Tong University School of Medicine. The study protocol was approved by the ethics committee of Shanghai Jiao Tong University School of Medicine. Written informed consent was obtained from all participants in this study. All the research was carried out in accordance with the provisions of the Declaration of Helsinki of 1975. None of these patients had received radiotherapy or chemotherapy prior to surgery. GSE110225 was an independent colorectal cancer patients’ GEO dataset. The clinical information of Cohort1, Cohort 2 and GSE11022 were listed in **Table S5**, **Table S6** and **Table S7**.

**Cell culture and treatment**

The human colorectal cancer cell lines DLD-1, RKO, HT29, SW480, HCT116, and human colorectal epithelial cell line FHC were purchased from American Type Culture Collection (ATCC). All the cell lines were cultured in the recommended growth medium added with 10% FBS (fetal bovine serum) in an atmosphere of 5% CO2 at 37℃. Cells were transfected with plasmids using FuGENE transfection reagent (Promega, Madison, WI) and transfected with siRNA using DharmaFECT 1 siRNA transfection reagent (Thermo Fisher Scientific, West Palm Beach, FL). Nonspecific siRNA or plasmids were used as negative controls. All siRNAs and primers sequences in this study were listed in **Table S8.**

**Generation of METTL3 knockout HCT116 cell lines (HCT116 METTL3-KO)**

Lentiviral-based CRISPR gene editing system (LentiCRISPR) was used to establish METTL3 knockout HCT116 cells. Genome engineering experiments using CRISPR-Cas9 systems were performed as described previously [1, 2]. Single guide RNAs (sgRNAs) targeting METTL3 were cloned into lentiCRISPR vector. HCT116 cells were transduced with lentiCRISPR-MELLT3 and selected with puromycin. The knockout cell clones were isolated by limited serial dilution. Monoclonal cell line was generated, and METTL3 knockout effect was confirmed using western blot and sequencing analysis. The sgRNA sequence targeting METTL3 was listed as follows: 5'-GGUGCUGUGGCAGAAAAGAA-3'.

**Adenovirus and plasmids construction**

The control plasmid, METTL3 overexpressing plasmids, METTL3-mut plasmids, HK2 overexpression plasmids, SLC2A1 overexpression plasmids were constructed by Generay Technologies (Shanghai, China). The control adenovirus, shControl adenovirus, METTL3 adenovirus, HK2 adenovirus, SLC2A1 adenovirus, shHK2 adenovirus, and shSLC2A1 adenovirus were constructed by Obio Technology Company (Shanghai, China).

**Total RNA extraction and real-time PCR**

Total RNA was extracted from CRC tissues and cell lines using trizol reagent (Takara, Japan). 1μg total RNA was reverse transcribed by PrimeScript RT Reagent Kit (Takara, Japan). PCR was performed using StepOnePlus real-time PCR system (Applied Biosystems, Foster City, CA) and 2^−ΔΔCt^ method was used to quantify the relative expression levels. β-actin was used as an internal control.

**Western blot**

Treated CRC cells were extracted by RIPA lysis buffer and quantified by BCA Protein Assay Kit (Thermo Fisher Scientific, West Palm Beach, FL). 40 μg of protein was separated by 10% SDS-polyacrylamide gels and then transferred to PVDF membranes (Biorad, Hercules, CA). The membranes were blocked with 5% BSA for 2 hours and incubated with primary antibody rabbit anti METTL3 (1:1,000 dilution, abcam, Cambridgeshire, UK), anti HK2 (1:1,000 dilution, CST, Boston, MA), anti GLUT1 (1:90,000 dilution, abcam, Cambridgeshire, UK), anti β-actin (1:20,000 dilution, Sigma, Louis, MO）at 4 ℃ overnight. Secondary antibodies (1:3,000 dilution, Kangcheng, China) were labeled with HRP. The ECL detection system (Biorad, Hercules, CA) was used for visualization.

**Cell proliferation and colony formation assay**

Cell proliferation was assessed by Cell Counting Kit-8 (Dojindo, Japan). Control and transfected CRC cells were planted into 96-well culture plates. Cell Counting Kit-8 was added to the cells at specific time points. After incubating for 2 hours away from light, the absorbance was measured by OD at 450 nm wavelength.

For the Colony formation assay, cells were transfected with indicated siRNA or plasmid. Treated cells were harvested, and 600 cells were seeded into 6-well culture plates. After 7 to 10 days of incubation, the cells were fixed with 4% paraformaldehyde, stained with 0.1% crystal violet washed with water and dried. Finally, the colonies were counted.

**Immunohistochemistry**

CRC tissue microarray sections were rehydrated and treated with hydrogen peroxide. Heat-mediated antigen retrieval was carried out by microwave with sodium citrate. The slides were incubated with indicated antibodies on a humidified box at 4℃, overnight. DAB substrate kit was used for the color-reaction, and hematoxylin was used for nucleus counterstaining. Protein expression was assessed according to the intensity and extent of staining at 200X under microscope. An intensity score from 0 to 3 (units of intensity, or UI) was given for each sample, and an average intensity score per tumor was calculated for each patient. The tissues with a final score ≤1.5 were classified as “Low Expression” and the tissues with a final score >1.5 were classified as “High Expression”.

**m^6^A dot blot**

The m^6^A dot blot assay was performed as previously described [3]. Polyadenylated mRNA was purified by GenElute^TM^ mRNA Miniprep Kit (Sigma, Louis, MO) from previously isolated total RNA. The poly(A)^+^ RNA samples were loaded to Hybond-N+ membrane (GE Healthcare, UK) and UV crosses with the nylon membrane. The membrane was then blocked with 5% nonfat milk for 1 hour and incubated with m^6^A antibody (Millipore, New Bedford, MA) at 4℃, overnight. After incubating with horseradish peroxidase-conjugated anti-mouse IgG, the membrane was visualized with the ECL detection system. The same amount of poly(A)^+^ RNA samples were spotted on the membrane and stained with 0.02% methylene blue (MB) in 0.3M sodium acetate (pH=5.2). The relative signal density of each dot was quantified by Image J software. The results of m^6^A level were shown in the form of relative m^6^A dot blot density normalized to methylene blue staining density.

**m^6^A quantification**

Total RNA was isolated from different cells by TRIzol reagent (Takara, Japan). Poly(A)^+^ RNA was purified using GenElute^TM^ mRNA Miniprep Kit (Sigma, Louis, MO). The global m^6^A levels in mRNA was measured with EpiQuik m^6^A RNA Methylation Quantification Kit (Colorimetric) (Epigentek, Farmingdale, NY) following the manufacturer’s protocol. 200 ng poly-A-purified RNA was used for each sample analysis.

**LC-MS/MS for quantification of m^6^A in mRNA**

200ng mRNA was digested with nuclease P1 (1U, Wako Pure Chemical, Osaka, Japan) in 25μl reaction buffer containing 20mM NH_4_OAc at 42℃ for 2 hours. Then the buffer was added with 3 μl 1M NH_4_HCO_3_ and alkaline phosphatase (1U, Sigma, Louis, MO) and incubated at 37℃ for another 2 hours. The samples were diluted to 50 μl, filtered through a 0.22μm-filter. Five-microlitre of the solution was loaded to a C18 reverse-phase column coupled online to Agilent 6410 triple-quadrupole LC-MS in positive electrospray ionization mode. Nucleosides were quantified by the nucleosides-to-base ion mass transitions of m/z 268 to 136 (A) and m/z 282 to 150 (m^6^A). The m^6^A/A ratio in mRNA was calculated by the standard curve obtained from pure nucleoside standards.

**MeRIP and** **MeRIP-qPCR**

Total RNA was extracted by Trizol reagent (Takara, Japan), and mRNA was purified using GenEluteTM mRNA Miniprep Kit (Sigma, Louis, MO). RNA fragmentation reagents (NEB, Hertfordshire, UK) were used to randomly fragment RNA. The specific anti-m^6^A antibody (NEB, Hertfordshire, UK) was applied for m^6^A immunoprecipitation. Anti-m^6^A antibody was pre-bound to Protein G magnetic beads in reaction buffer for 30 minutes. The fragmented mRNA was incubated with m6A-antibody-bound protein G magnetic beads at 4℃ for 1 hour and washed with low salt reaction buffer and high salt reaction buffer. m^6^A-antibody-bound RNA was extracted from the Dynabeads using Buffer RLT (Qiagen, Hilden, German) and further incubated with Dynabeads MyOne Silane (Life Technologies, West Palm Beach, FL). The RNA and Dynabeads mixture were precipitated with 100% ethanol washed with 70% ethanol and then re-suspend with nuclease-free water. The supernatant was carefully collected after the beads were pulled to the side of the tube by a magnetic field. Real-time PCR was carried following m^6^A-IP to quantify the changes to m^6^A methylation of a certain target gene.

**Luciferase assay**

The fragments of HK2-5’UTR, HK2-3’UTR and SLC2A1-3’UTR containing the wild-type m^6^A motifs as well as mutant m^6^A motifs (m^6^A was replaced by T) were synthesized at Generay Technologies (Shanghai, China). The wild-type and mutant HK2-5’UTR fragments were inserted into the upstream of pGL3-basic firefly luciferase vector, while the wild-type and mutant HK2-3’UTR, SLC2A1-3’UTR fragments were subcloned into the downstream of pMIR-REPORT firefly luciferase vector. CRC cells were seeded in 96-well plates and co-transfected with 500ng wild-type or mutant HK2-5’UTR (or HK2 3’UTR or SLC2A1-3’UTR), 100ng pRL-TK plasmid (Renilla luciferase) and 5μL METTL3-siRNA. The relative luciferase activity was detected using the Dual-Luciferase Reporter Assay System (Promega, Madison, WI) 24 hours later. Firefly luciferase activity and Renilla luciferase activity were measured using FLUOstar Omega (BMG LABTECH, Offenburg, Germany). The results were shown in the form of relative firefly luciferase activity normalized to Renilla luciferase activity. All the experiments were repeated for three times, and three replicates were conducted for each group.

**RNA stability assay**

CRC cells with or without METTL3 knockdown were treated with Actinomycin D for 0h, 3h, and 6h at a final concentration of 5 μg/mL. Total RNA was extracted, and real-time PCR was conducted to quantify the relative level of HK2 or SLC2A1 mRNA. The degradation rate and half-life of HK2 or SLC2A1 mRNA were estimated according to the published paper. Briefly, the degradation rate of mRNA (*K*_decay_) was calculated by the following equation:

ln(C/C_0_)=- *K*_decay_*t*

*t* is the transcription inhibition time, and *C* is the mRNA level at the time *t*. *C_0_* is the level of mRNA at 0 hour in the equation, which means the mRNA level before decay starts. Thus, the mRNA half-time (t*_1/2_*) can be calculated by the equation:

In(1/2)= - *K*_decay_*t_1/2._*

**Glucose uptake**

Glucose Uptake Colorimetric Assay Kit (abcam, Cambridgeshire, UK) was used to determine glucose uptake according to the manufacturer’s protocols. CRC cells were seeded into 6-well plates and transfected with indicated constructs and incubated for 24 hours. The transfected cells were harvested, and the cell number was calculated. 1x10^4^ cells were planted into 96-well cell culture plates and incubated at 37℃, overnight. Next day cells were starved for glucose for 2 hours. After incubating with 100μl Krebs-Ringer-Phosphate-HEPES for 40 minutes, 10μl 10mM 2-DG was injected into each well and incubated for 20 minutes. And then cells were collected with extraction buffer and used for determination of glucose uptake. The glucose uptake was measured by OD at 412 nm wavelength.

**Lactate production and hexokinase activity assay**

L-Lactate Assay kit (Colorimetric) was used to measure the lactate production (abcam, Cambridgeshire, UK) according to the manufacturer’s protocols. The transfected cells were planted into 96-well cell culture plates and incubated at 37℃, overnight. After starvation for 2 hours, the supernatant was collected for measurement of lactate production. The lactate production levels were measured at 450 nm in a microplate reader. Hexokinase Assay Kit (Colorimetric) was used to detect hexokinase activity (abcam, Cambridgeshire, UK) according to the manufacturer’s protocols. CRC cells were seeded into 6-well plates and transfected with indicated constructs and incubated for 48 hours. 1x10^6^ transfected cells were harvested and homogenized with hexokinase assay buffer. After centrifuged for 5 minutes, the supernatant was collected for measurement of hexokinase activity.

**Seahorse metabolic analysis**

ECAR and OCR were measured using Seahorse XF Glycolysis Stress Test Kit and Seahorse XF Cell Mito Stress Test Kit (Agilent Technologies, Palo Alto, CA). CRC cells were seeded into the 96-well cell culture plates in medium with 10% FBS and incubated at 37℃ overnight and then the cells were used for measurement of ECAR and OCR. After measurement of baseline concentration, glucose, oligomycin, and 2-DG were sequentially added into each well for ECAR measurement. Oligomycin, FCCP (p-trifluoromethoxy carbonyl cyanide phenylhydrazone), and Antimycin A & Rotenone were sequentially injected into each well for OCR measurement. Seahorse XF-96 Wave software was used to analyze the data.

**Mass spectrometric metabolomics analysis**

CRC cell lines HCT116 WT and METTL3-KO cells were collected and added with 1mL pre-cooled methanol/acetonitrile/water (2:2:1, v/v/v). The samples were vortexed, sonicated at 4 °C for 20 minutes and incubated at -20 °C for 1 hour to precipitate proteins. The mix was centrifuged at 14000 rcf for 10 minutes at 4 ℃ and dried under vacuum. For mass spectrometry, the dried samples were dissolved in 100 μL of acetonitrile-water solution (1:1, v/v) and centrifuged at 14,000 rcf for 10 min at 4 °C. Electrospray ionization was conducted using Agilent 1290 Infinity chromatography system and QTRAP 5500 (AB SCIEX) mass spectrometer.

**RNA immunoprecipitation**

RNA Immunoprecipitation (RIP) assays were conducted using the Magna RIP Kit (Millipore, New Bedford, MA) according to the manufacturer’s protocols. Cells were prepared using RIP lysis buffer and the RNA-protein complexes were immunoprecipitated using anti-IGF2BP1 antibody (CST, Boston, MA), anti-IGF2BP2 antibody (PROTEINTECH, Rosemont, IL), anti-IGF2BP3 antibody (PROTEINTECH, Rosemont, IL) and normal rabbit IgG. The co-precipitated RNAs were purified using phenol:chloroform:isoamyl alcohol and subjected to reverse transcription-PCR or real-time PCR analysis. A control amplification was carried out on the input RNA before immunoprecipitation.

***In vivo* xenograft model**

To illustrate the effect of METTL3 on tumor growth *in vivo*, 4-week-old male BALB/c nude mice were purchased from Experimental Animal Center of SIBS. HCT116 WT or METTL3-KO cells and DLD1 cells were injected into the right flank of mice subcutaneously to establish the CRC xenograft model. Seven days after inoculation, the mice were injected with indicated adenovirus by multipoint intratumoral injection every two days. 2DG was administered at 800mg/kg body weight by intraperitoneal route every other day. Tumor volume (mm^3^) was assessed by the formula: Tumor volume (mm^3^) = longer diameter x shorter diameter^2^/2.

**^18^F-FDG PET imaging**

Mice were fasted for 8 hours and injected with approximately 250 μCi of ^18^F-FDG via lateral tail vein (the exact dose was calculated by measuring the syringe before and after injection). Mice were maintained in cages at room temperature for 1 hour and anesthetized with isoflurane. Mice were placed on a warm pad in the prone position, followed by micro-PET and micro-CT imaging. ^18^F-FDG uptake was quantified by drawing region of interest (ROI) using AMIDE software and plotting maximum uptake values (SUV_max_).

**High-throughput sequencing**

For RNA sequencing, each sample was cleaned up on a RNeasy Mini Column (QIAGEN, Hilden, Germany), treated with DNase, and analyzed for quality on an Agilent 2100 Bioanalyzer. Samples were run on an Illumina HiSeq 3000 for 2X150-bp paired-end sequencing. The RNA-seq data analysis was performed according to the TopHat- HTSeq-DeSeq2 frame[4] as described in our previous study. Briefly, reads were mapped to the human genome (hg19) using TopHat v2.0.11 [5] (http://tophat.cbcb.umd.edu) with the default options with a TopHat transcript index built from Ensembl_GRCh37. Count files of the aligned sequencing reads were generated by the htseq-count script from the Python package HTSeq with union mode, using the GTF annotation file [6]. The read counts from each sequenced sample were combined into a count file, which was subsequently used for the differential expression analysis. Differential analyses were performed to the count files using DESeq2 packages, following standard normalization procedures [7]. Genes with less than five total counts in both conditions were removed from further analysis. The RNA sequence data have been deposited in NCBI’s Gene Expression Omnibus (GEO, http://www.ncbi.nlm.nih.gov/geo/) and are accessible through GEO Series accession number GSE130012. For m^6^A sequencing, samples were sequenced by Illumina HiSeq 3000 with single-end 150-bp read length. All reads were mapped to human genome hg19 by tophat v2.0.11 with default settings. The m^6^A level changes for METTL3-KO / WT were calculated by using exomePeak [8].

**References**

1. Ran FA, Hsu PD, Wright J, Agarwala V, Scott DA, Zhang F. **Genome engineering using the CRISPR-Cas9 system.** *Nat Protoc.* 2013; **8:**2281-308.

2. Sato H, Kosaka W, Matsuda R, Hori A, Hijikata Y, Belosludov RV, Sakaki S, Takata M, Kitagawa S. **Self-accelerating CO sorption in a soft nanoporous crystal.** *Science.* 2014; **343:**167-70.

3. Li Z, Weng H, Su R, Weng X, Zuo Z, Li C, Huang H, Nachtergaele S, Dong L, Hu C, et al. **FTO Plays an Oncogenic Role in Acute Myeloid Leukemia as a N(6)-Methyladenosine RNA Demethylase.** *Cancer Cell.* 2017; **31:**127-41.

4. Anders S, McCarthy DJ, Chen Y, Okoniewski M, Smyth GK, Huber W, Robinson MD. **Count-based differential expression analysis of RNA sequencing data using R and Bioconductor.** *Nat Protoc.* 2013; **8:**1765-86.

5. Kim D, Pertea G, Trapnell C, Pimentel H, Kelley R, Salzberg SL. **TopHat2: accurate alignment of transcriptomes in the presence of insertions, deletions and gene fusions.** *Genome Biol.* 2013; **14:**R36.

6. Anders S, Pyl PT, Huber W. **HTSeq--a Python framework to work with high-throughput sequencing data.** *Bioinformatics.* 2015; **31:**166-9.

7. Love MI, Huber W, Anders S. **Moderated estimation of fold change and dispersion for RNA-seq data with DESeq2.** *Genome Biol.* 2014; **15:**550.

8. Meng J, Lu Z, Liu H, Zhang L, Zhang S, Chen Y, Rao MK, Huang Y. **A protocol for RNA methylation differential analysis with MeRIP-Seq data and exomePeak R/Bioconductor package.** *Methods.* 2014; **69:**274-81.
