# Supplementary figures and images for: m6A-dependent glycolysis enhances colorectal cancer progression
Source: Mol Cancer. 2020 Apr 3;19:72. doi: 10.1186/s12943-020-01190-w (PMC7118901; doi:10.1186/s12943-020-01190-w)

Figure S1

a

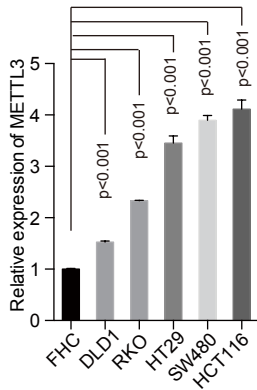

b

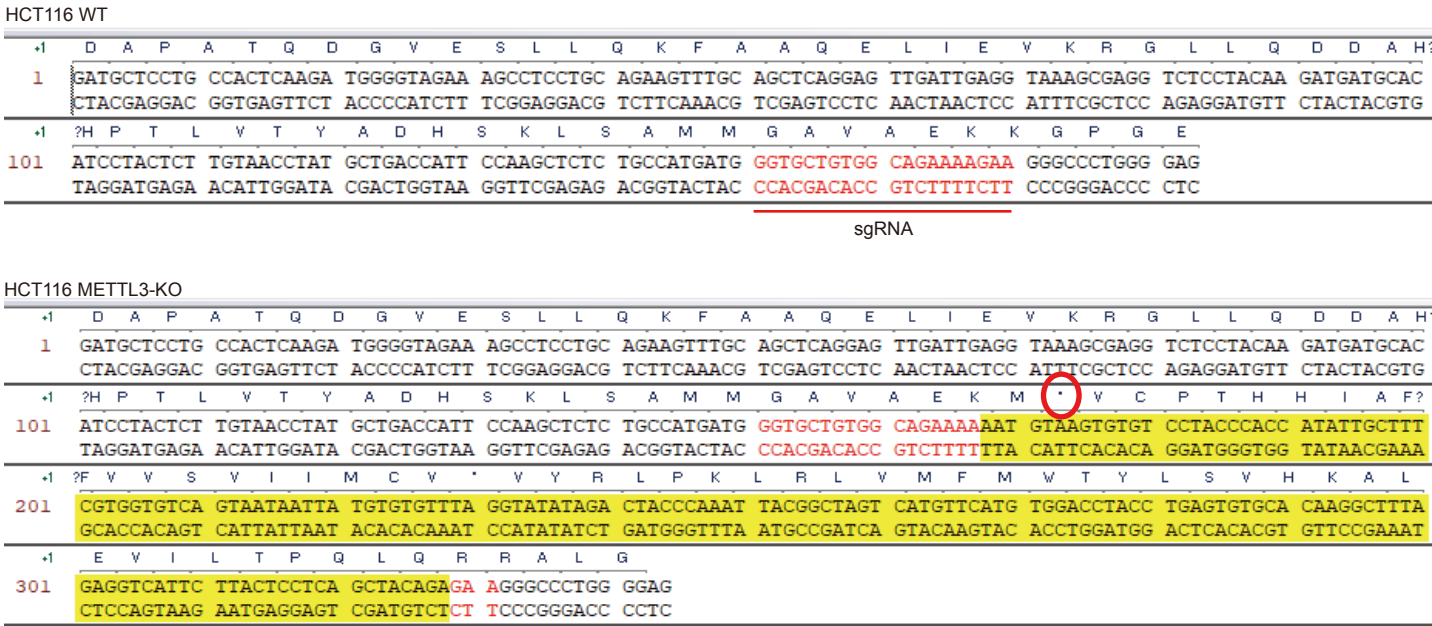

c

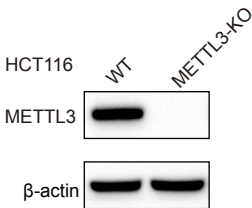

d

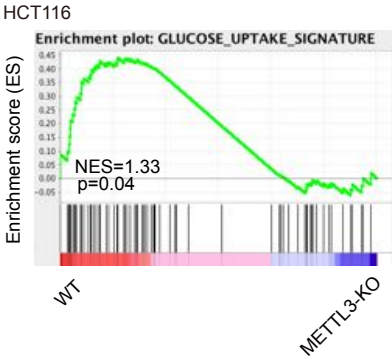

Figure S2

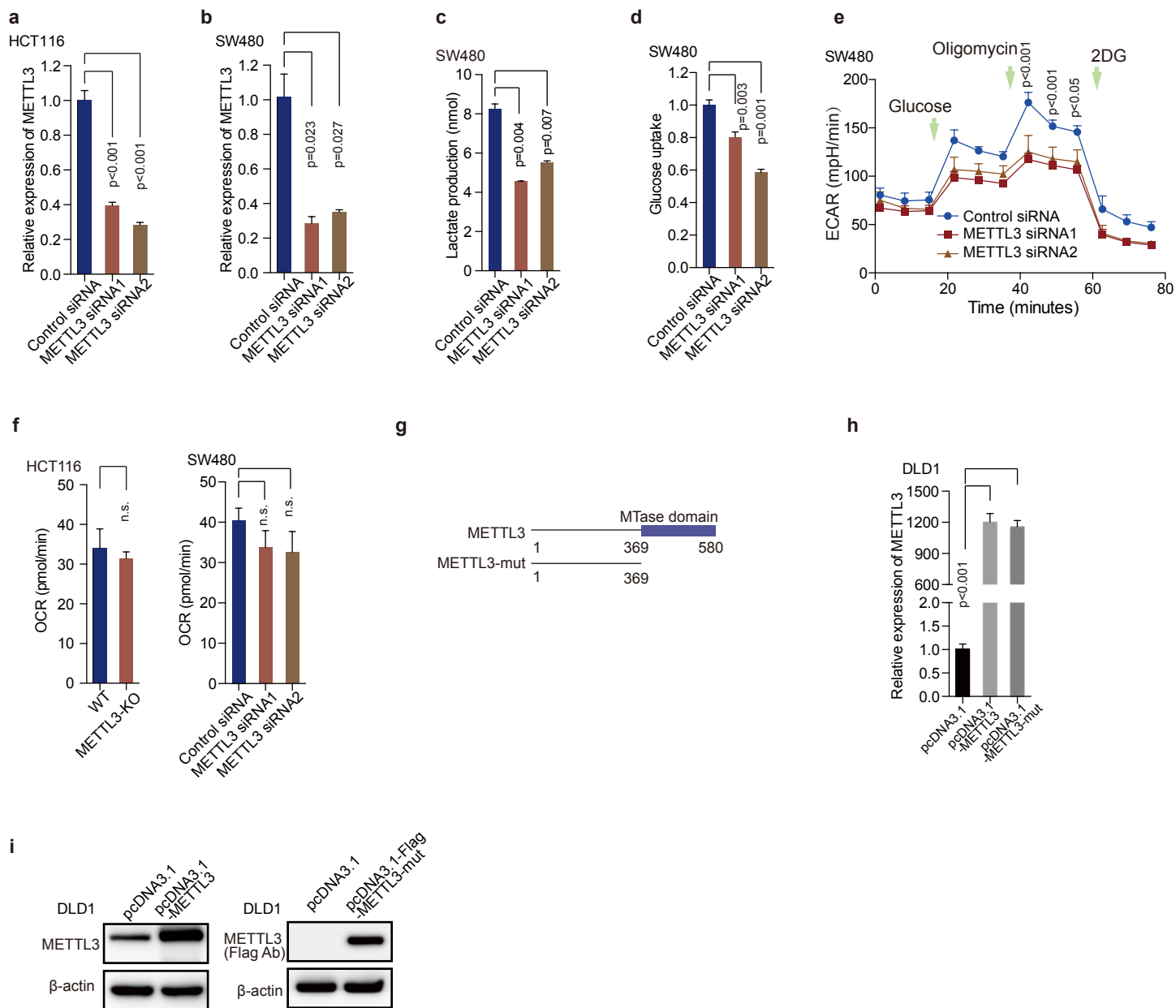

Figure S3

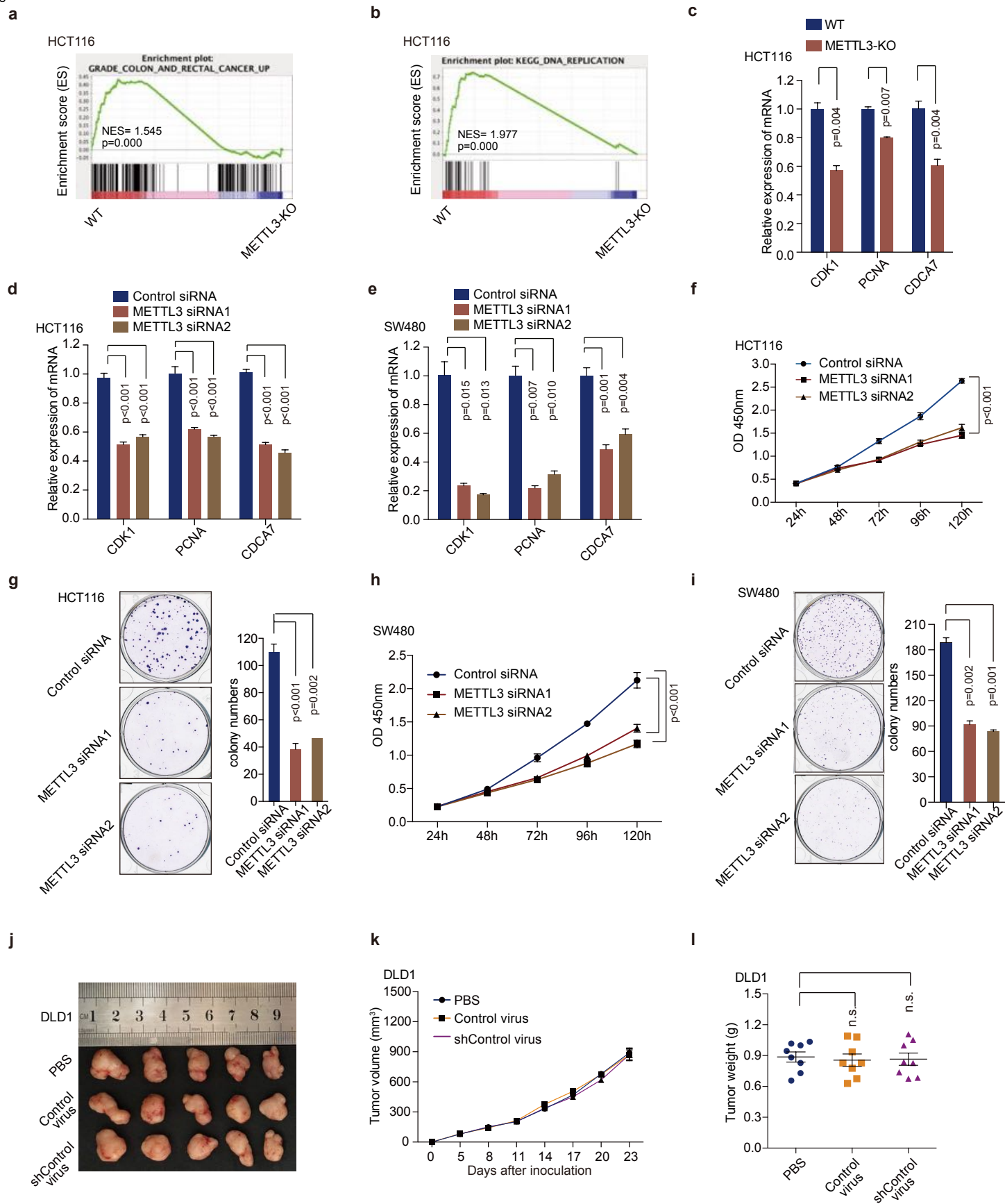

Figure S4

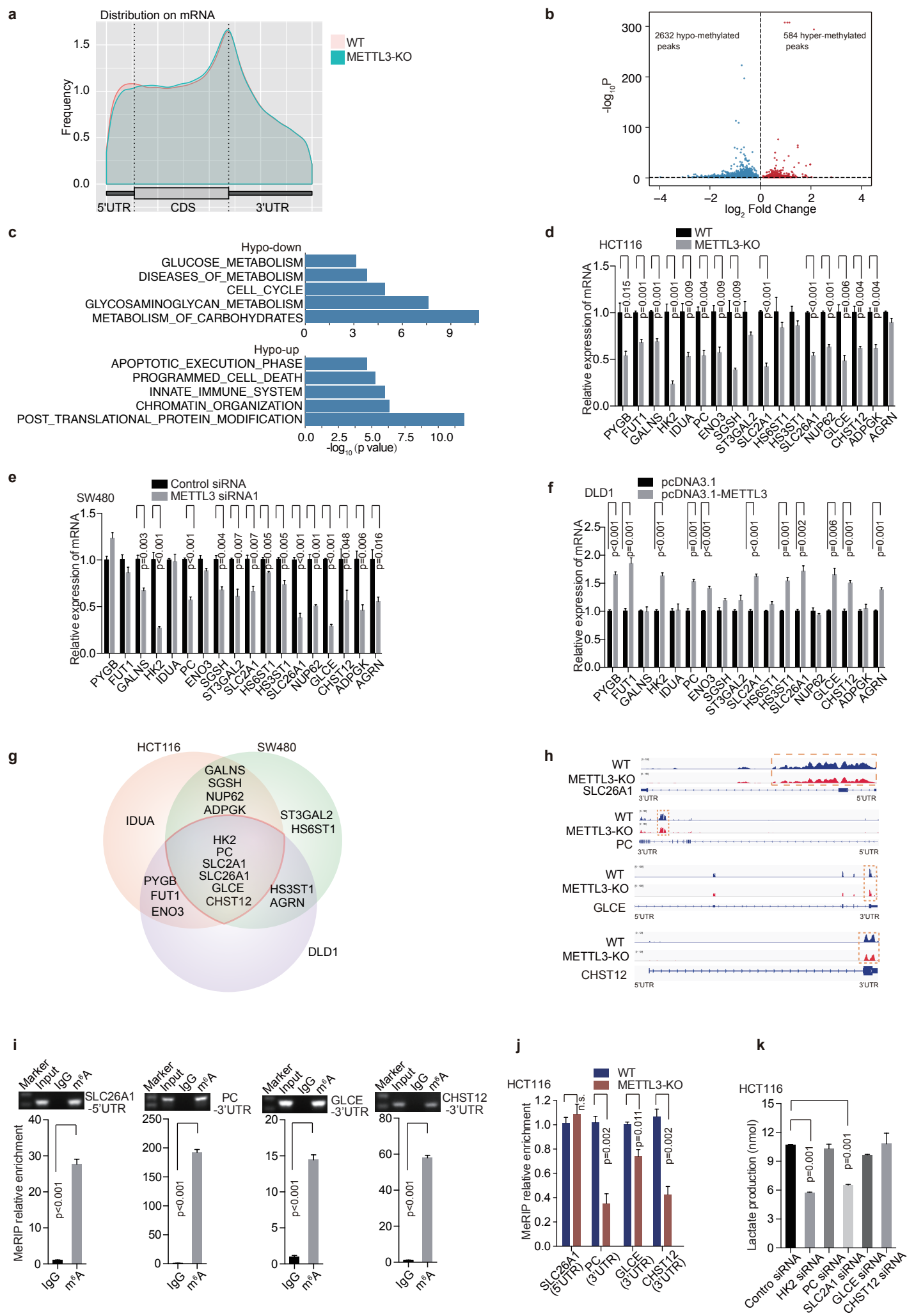

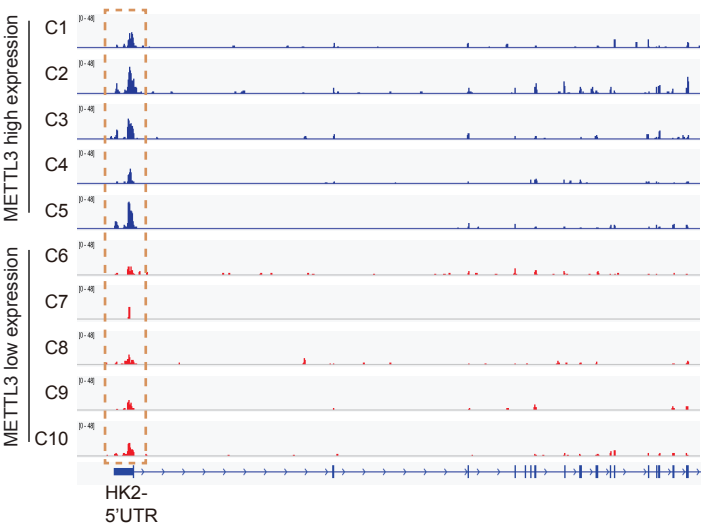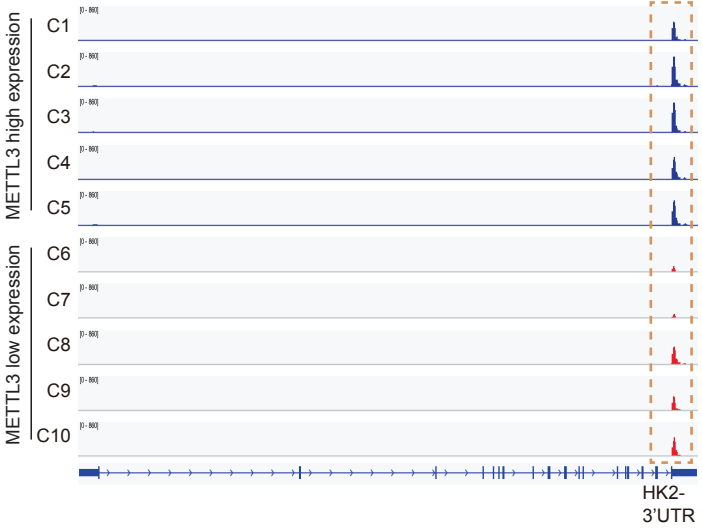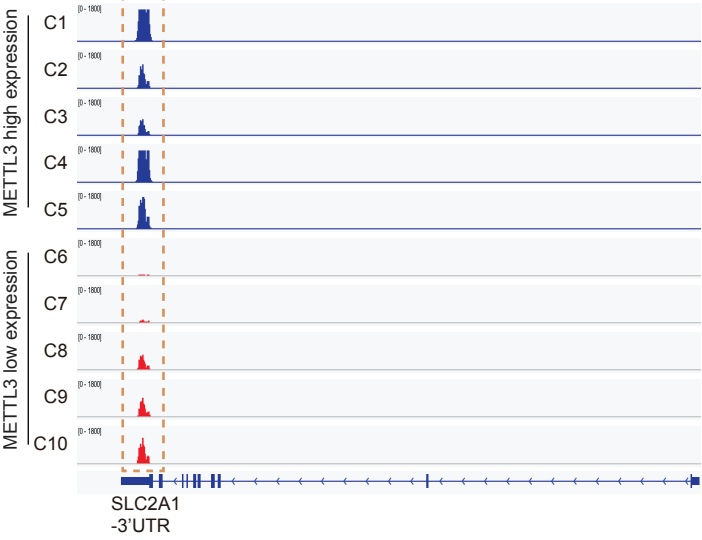

Figure S5

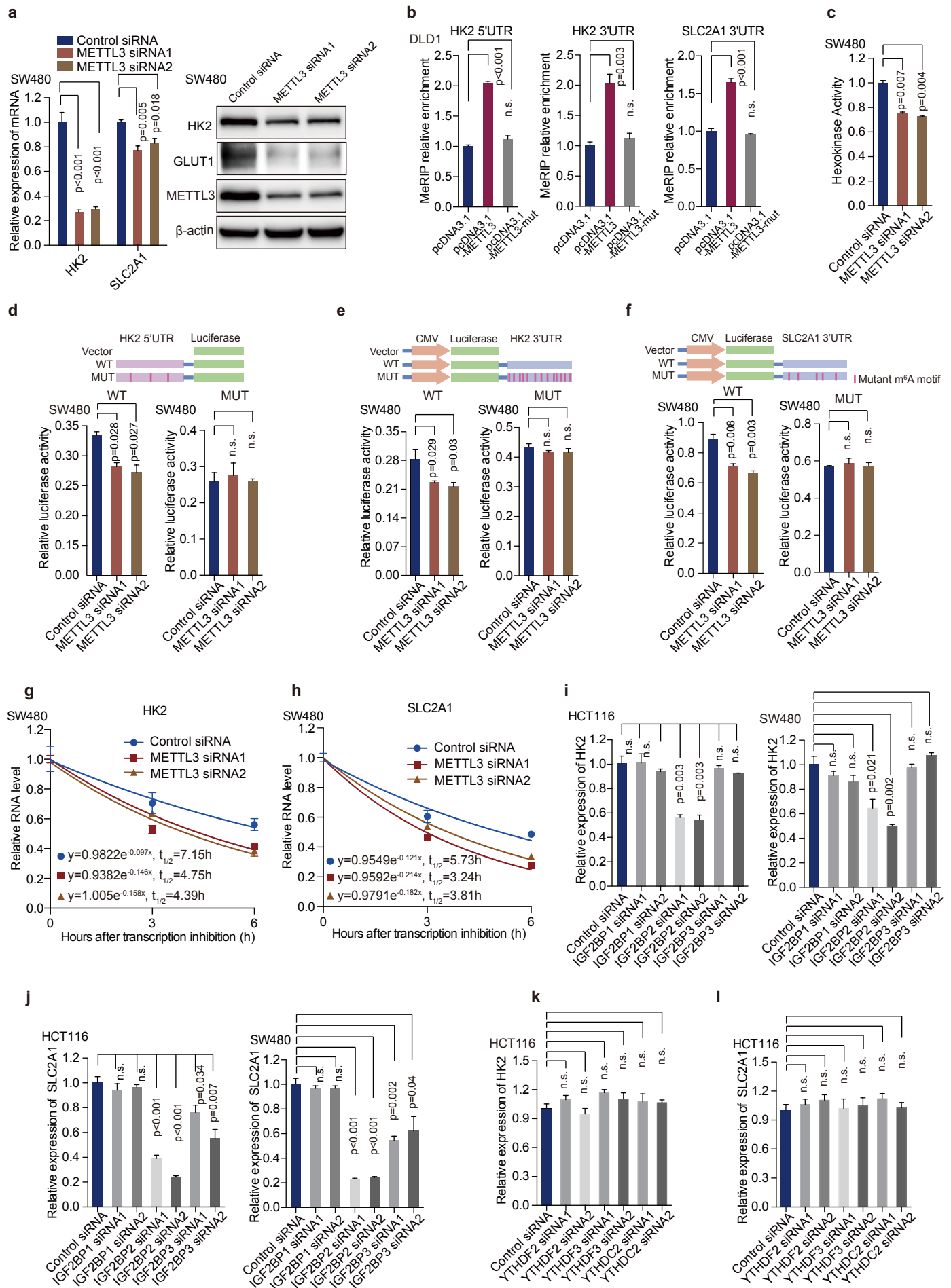

Figure S6

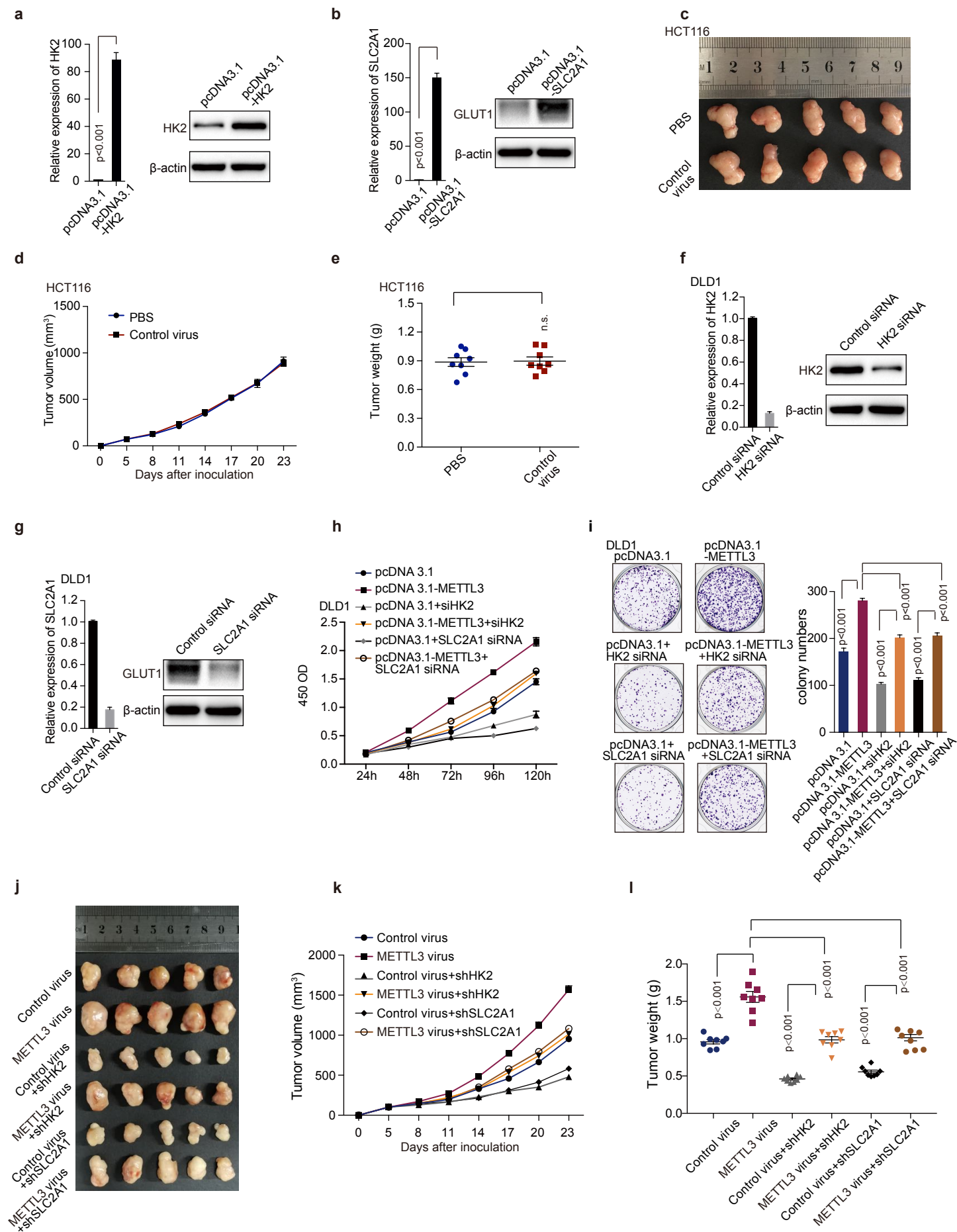

Figure S7

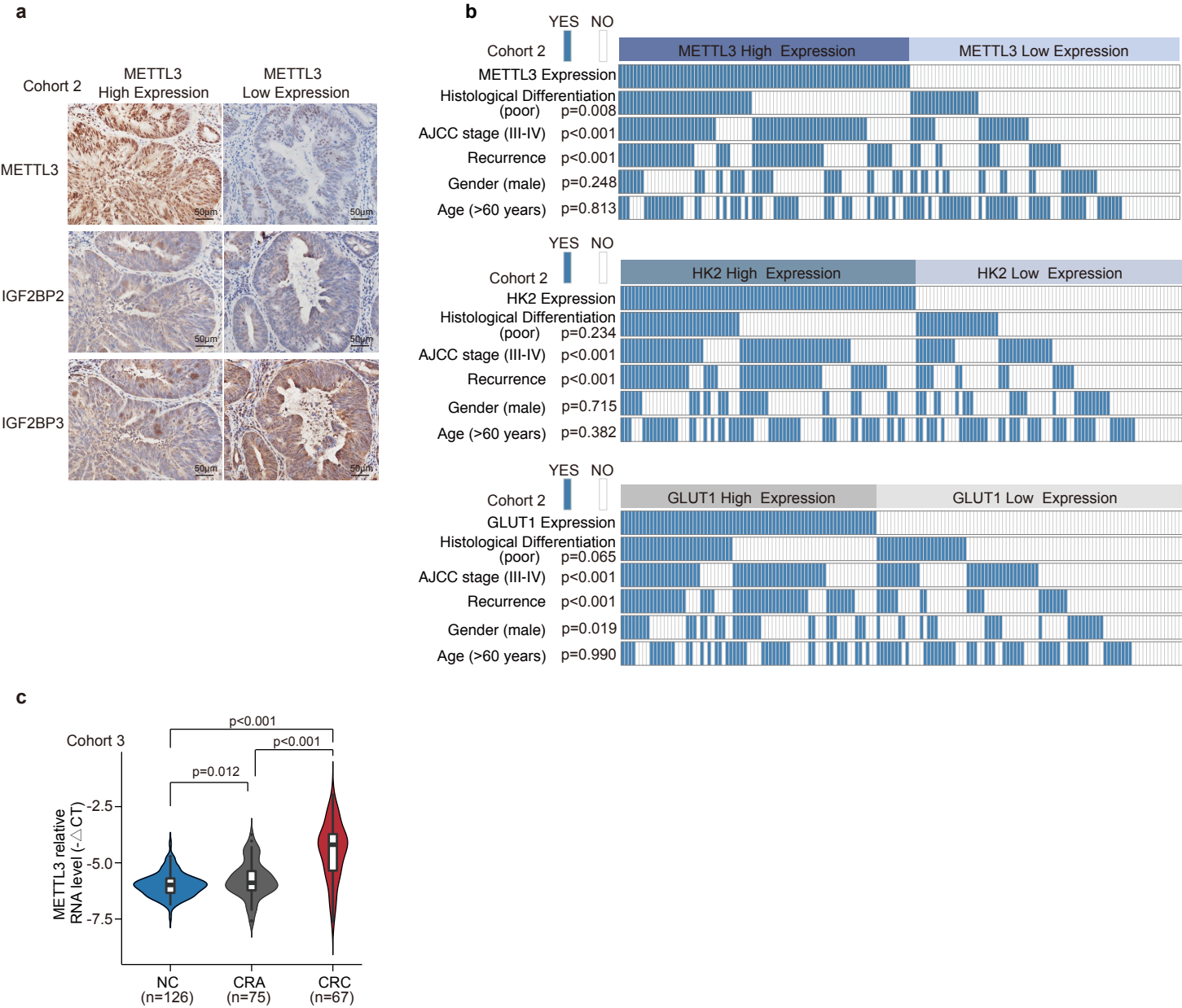

Supplement: Supplementary file 3 — Additional file 3 Figure S1. METTL3 is closely correlated with glycolytic metabolism in CRC. (a) The relative expression of METTL3 was measured by real-time PCR in normal colonic epithelial cell line FHC and CRC cell lines, n = 3, nonparametric Mann–Whitney test. (b) Schematic diagram of METTL3 genomic sequence in HCT116 wild-type (WT) and METTL3-knockout (METTL3-KO) cells. Red label, target sgRNA sequence; Yellow label, A 161 bp DNA fragment was inserted in the cutting site; Red circle, a premature stop site was generated after insertion of 161 bp DNA fragment. (c) The knockout efficiency of METTL3 was confirmed by Western blot. (d) GSEA analysis was conducted to identify the differential gene profiles between HCT116 METTL3-KO and WT cells. (WT, wild type; METTL3-KO, METTL3-knockout). Figure S2. METTL3 drives glycolytic metabolism in CRC. (a-b) The knockdown efficiency of METTL3 siRNA1/2 was evaluated in HCT116 (a) and SW480 (b) cells, n = 3, nonparametric Mann–Whitney test. (c-e) Lactate production (c), glucose uptake (d) and ECAR (e) were measured after transfection of control siRNA and METTL3 siRNA1/2 in SW480 cells, n = 3, nonparametric Mann–Whitney test. (f) OCR was measured in HCT116 WT and METTL3-KO cells (left). OCR was measured in SW480 cells transfected with control siRNA and METTL3 siRNA1/2 (right). (g) Schematic domain structures of METTL3. METTL3 MTase (AA residues 369–580). (h-i) Real-time PCR (h) and Western blot assay (i) were performed to detect METTL3 expression after transfection with pcDNA3.1-METTL3 and pcDNA3.1-METTL3-mut in DLD1 cells. (WT, wild type; METTL3-KO, METTL3-knockout). Figure S3. METTL3 is an oncogenic gene in colorectal cancer. (a-b) GSEA analysis was conducted to identify the differential gene profiles between HCT116 METTL3-KO and WT cells. (c) The expression of CDK1, PCNA and CDCA7 were detected in HCT116 WT and METTL3-KO cells, n = 3, nonparametric Mann–Whitney test. (d-e) The expression of CDK1, PCNA and CDCA7 were detected aft [file 12943_2020_1190_MOESM3_ESM.pdf]
